# Ondansetron derivatives as potential PTP1B inhibitors for treating type 2 diabetes mellitus: *in silico*, *in vitro*, and *in vivo* analysis

**DOI:** 10.3389/fphar.2026.1796359

**Published:** 2026-06-03

**Authors:** Fawad Naeem, Wei Jiang, Mustafeez Mujtaba Babar, Syed Haroon Hussain, Zaman Ashraf, Jiayi Sun, Sohaib Zafar, Qiang Liu

**Affiliations:** 1 Riphah Institute of Pharmaceutical Sciences, Riphah International University, Islamabad, Pakistan; 2 Shifa College of Pharmaceutical Sciences, Shifa Tameer-e-Millat University, Islamabad, Pakistan; 3 Central Hospital of Dalian University of Technology, Dalian, China; 4 Department of Chemistry, Rawalpindi Women University, Rawalpindi, Pakistan; 5 Faculty of Medicine, Dalian University of Technology, Dalian, China

**Keywords:** computational studies, *in vitro*/*in vivo* studies, ondansetron derivatives, PTP1B inhibition, type 2 diabetes

## Abstract

**Introduction:**

Type 2 diabetes has become one of the most common causes of human deaths. Key factors that contribute to the progression of this disease include a sedentary lifestyle, high-fat diet, and genetic abnormalities. Enzyme PTP1B is an important target for researchers as it plays a vital role in performing normal metabolic functions of the human body. Metabolic diseases such as obesity, type 2 diabetes, and cardiovascular complications are due to PTP1B imbalance. It is evident that the overproduction of PTP1B is a prominent factor contributing to the onset and progression of type 2 diabetes.

**Methods:**

Multiple attempts have been made in recent years to synthesize PTP1B inhibitors but, in most cases, complications were associated with the selectivity and toxicity of the synthesized analogs, which resulted in the failure of their clinical trials. This study comprises a series of ondansetron derivatives, which were developed by employing a one-pot (reductive amination) reaction between ondansetron and substituted anilines to synthesize primary amines as final products. These final products (ondansetron derivatives) were analyzed as PTP1B inhibitors to study the role of enzyme PTP1B in lowering blood glucose levels in conditions such as type 2 diabetes. These novel compounds can provide valuable insights into the development of improved treatment options for diabetes.

**Results:**

The synthesized PTP1B inhibitors were subject to *in silico*, *in vitro*, and *in vivo* analysis that revealed encouraging results. The computational data of the selected ligand FN-06 showed highest binding affinity, with the protein PTP1B (1T48) having a docking score of −7.317 kcal/mol. The indicators of the simulation also reflected the stability of the said ligand. Similarly, the results of an *in vitro* inhibition assay further confirmed that the synthesized compound FN-10 showed direct inhibition of the enzyme PTP1B. Furthermore, animal studies demonstrate the hepatoprotective and anti-diabetic action of the selected compounds FN-06 and FN-10 along with the parent compound ondansetron.

**Discussion:**

The findings of this study suggest that compounds FN-06 and FN-10 may act as lead structures to design potent derivatives, which have antidiabetic activity with hepatoprotective effects.

## Graphical Abstract

**Figure:**
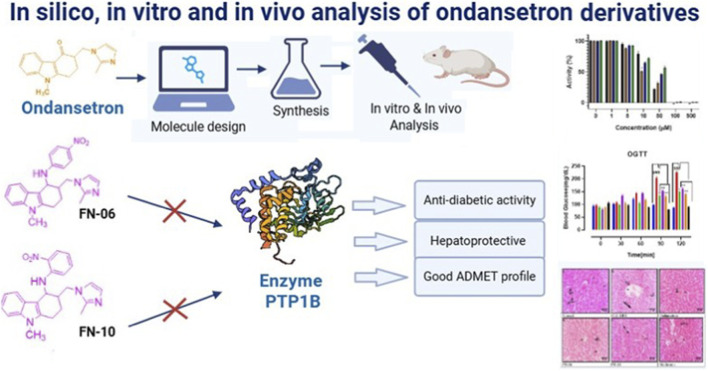

## 1 Introduction

The impaired regulation of protein tyrosine phosphatase 1B (PTP1B) in the human body is a leading cause of numerous abnormal conditions, including obesity, diabetes, and cancer. Protein tyrosine phosphatases (PTPs) have gained a much attention as a molecular target in these conditions (Cohen and Alessi, 2013). Both PTPs and protein tyrosine kinases (PTKs) are primarily involved in the regulation of the tyrosine phosphorylation levels that are essential to the normal functioning of cellular processes that include differentiation, growth, apoptosis, and even survival (Sharma et al., 2020). PTP1B negatively regulates leptin and insulin-signaling pathways that are involved in controlling glucose levels, body weight, and metabolism. By the direct phosphorylation of the insulin receptor (IR) and insulin receptor substrates (IRS), PTP1B downregulates insulin signaling while it affects leptin signaling through the JAK2-mediated deregulation of the STAT3 pathway (Villamar-Cruz et al., 2021; Teimouri et al., 2022). This pathway leads to altered glucose uptake, metabolite regulation, apoptosis inhibition, and vasodilation, potentially contributing to the management of diabetes mellitus and other metabolic conditions.

Two main binding sites are present in enzyme PTP1B: the catalytic and the phosphotyrosine binding sites. It is evident that the ligands designed specifically against these binding sites not only increase activity but also produce inhibitors that are more selective between TCPTP and PTP1B (Liu et al., 2012; Ottanà et al., 2012). Depending upon different binding properties, various inhibitors were synthesized with respect to TCPTP and PTP1B. These inhibitors had a close structural resemblance and showed identical binding affinity with PTP1B. They also showed high-quality selectivity that varied between TCPTP and PTP1B.

One such nucleus that effectively binds and potently inhibits several biomolecules is the carbazole nucleus, and it has gained in importance since it has been demonstrated to develop compounds that show anti-diabetic activity. It has been evident that heterocyclic compounds with a carbazole nucleus tend to produce anti-diabetic activity in studies led by Zhang et al. (2018) and Iqbal et al. (2017). A number of strategies have been employed to make the medicinal compounds more active. Of these, bio-isosteric replacement strategy not only makes the molecule more potent but also increases its bioavailability (Liu et al., 2016). An increase in bioavailability and potency was reported, aimed at the PTP1B catalytic pocket, in the ZINC02765569 compound when the carboxylic group (-COOH) was replaced by the tetrazole group (Lima and Barreiro, 2005).

Heterocyclic derivatives including tetrazoles, thiazolidine dione derivatives, thiophene derivatives, benzotriazoles, isoxazoles, and triaryl sulfonamides have also been reported to possess PTP1B inhibitory activities (Wang et al., 2014; Meng et al., 2016). Similarly, studies suggest that by targeting the thiophene moieties in a heterocyclic compound, the binding affinities of various heterocyclic compounds can be enhanced against the enzyme PTP1B (Moretto et al., 2006; Wilson et al., 2007; Sztanke et al., 2015; Buduma et al., 2016).

Flavonoids of natural origin, having low toxicity profile and strong blood glucose lowering properties, are known to be potent PTP1B inhibitors (Nguyen et al., 2011; Jung et al., 2017). Flavonoids that have nitrogen in their heterocyclic ring are known to possess strong activity. It has also been reported that the azole ring in flavonoid structure possesses inhibitory activity against enzyme PTP1B (Liu et al., 2012; Ong et al., 2012).

Based on a similar, targeted derivatization approach, an amine group was incorporated. The ondansetron derivatives were synthesized by employing a reductive amination (one-step) reaction to form amines, which have diverse pharmaceutical applications as drug molecules due to their favorable and encouraging chemical properties. Some of their important characteristics include basicity, proficient receptor binding, lipophilicity, water solubility, nucleophilicity, and chemical stability. Here, we report the design and synthesis of a series of new ondansetron derivatives. Several substituted anilines were introduced to the carbazole nucleus of ondansetron. These derivatives were characterized using physical and structural elucidation. Their medicinal properties were established through a series of *in vitro* and *in vivo* experiments. Their antidiabetic potential was also determined by developing a streptozotocin (STZ)-induced high-fat-diet model of diabetes and studying their morphological, histological, and hematological properties.

## 2 Methodology

### 2.1 *In silico* analysis

#### 2.1.1 Ligand design strategy

**FIGURE 1:**
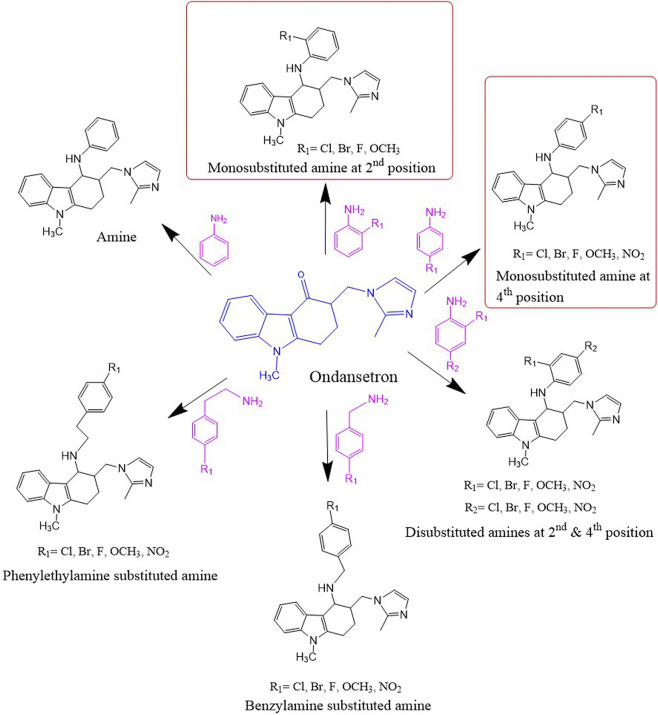
Ligand design strategy for the synthesis of ondansetron derivatives FN-01 to F-11 incorporating mono-substituted anilines at positions 2 and 4 of the phenyl ring.

Substituted anilines were used to design a series of ondansetron derivatives based on the selection of different functional groups wherein each group possessed unique chemical properties and reactivity. This included the incorporation of mono-substituted anilines at positions 2 and 4 of the phenyl ring, disubstituted anilines having substituents at positions 2 and 4 of the benzene ring, benzylamines, and phenylethylamines. This study designed and synthesized mono-substituted anilines at positions 2 and 4 and evaluated them as PTP1B inhibitors (Figure 1).

### 2.2 Molecular docking analysis

Computational analysis was performed to determine the interaction between ondansetron and its derivatives, with enzyme PTP1B having PDB ID:1T48 already reported by Shinde et al. (2018). Ondansetron and its derivatives were also analyzed against 5-HT_3_ or the serotonin receptor, which is a known target for the drug. The Glide module of Maestro release 2022 (Schrodinger, LLC, New York, United States) was used for molecular docking studies. A series of carbazole derivatives (ligands) was prepared using the 2D sketcher of the ligand preparation/library design module of Maestro (Figure 1). The structures of the target proteins PTP1B and serotonin receptor having PDB ID’s 1T48 and 6W1M were obtained from the RCSB Protein Databank (http://www.rcsb.org) in PDB format. After subjecting the protein structure to the process of cleaning, incorporating the missing atoms, refining hydrogen bonds, and adding charges and right bond order, the files proceeded to docking. The ligand molecules were subjected to the LigPrep tool by which they were converted into the optimized 3D models favorable for detailed computational studies. A receptor grid is a cubic space present around the active site of the selected protein. Standard grid dimensions of 30 Å × 30 Å × 30 Å for the outer box and 10 Å × 10 Å × 10 Å for the inner box were selected. After grid generation, the protein and ligand molecules proceeded to the molecular docking procedure that further led to analysis, which obtained the binding affinity and interaction modes of the docking complexes. Docking scores were used to rank different ligand conformations and orientations to identify the most likely binding poses, out of which the best ligand was selected for further study.

### 2.3 Molecular dynamics simulation

Molecular dynamics simulation studies were conducted to determine the protein and protein–ligand complexes in physiological conditions. In the MD simulation study, the movement of atoms was calculated for a specific time span utilizing Newton’s classical equation of motion. The MD simulation study was conducted for the ligand molecule (FN-06) exhibiting the maximum binding affinity for PTP1B (PDB ID:1T48). This study utilized the Desmond module of the Schrodinger software in which the ligand–protein complex was subjected to 200 ns MD simulations to assess the stability of the selected ligand at the active site of the protein. A simulation system was prepared by utilizing the system builder tool present in the Desmond module. An adequate quantity of Na^+^ and Cl^−^ ions were added to achieve system neutralization. Single-point charge (SPC) analysis was determined using a solvation model. The protein–ligand complex was subjected to the periodic boundary conditions (PBCs) pertaining to the orthorhombic simulation box. A buffer space of 10 Å was kept between the box and the solute. Minimization equilibration was carried out prior to running the final MD simulation job. A Nosé–Hoover chain thermostat was used to maintain the temperature at 300 K and pressure at 1.0132 bar. Analysis of the protein–ligand complex was carried out by observing the MD trajectories present in the built-in Maestro module (Mishra et al., 2021).

### 2.4 ADMET profile

The physicochemical properties of ligands were explored to understand the pharmacokinetic profile and select the most appropriate ligand. The important parameters involved in the drug development process are highlighted in the ADMET profile and include drug-likeness, bioavailability, ability to penetrate the blood–brain barrier, percentage oral absorption, plasma–protein binding, dermal penetration, and IC_50_ values. The online tool SwissADME was used to determine the pharmacokinetic profile of the ligands. The 2D structures of the ligands were drawn, and the SMILES files were extracted from them. Finally, the program was run to obtain the complete pharmacokinetic profile.

#### 2.4.1 Protein tyrosine phosphatase 1B inhibition assay

A PTP1B inhibitor Screening Assay Kit (ab 139465, Abcam) was employed to determine the inhibitory *in vitro* activity of the minimum inhibitory concentration-1 against enzyme PTP1B. The assay is designed to evaluate the inhibitory effect of MIC (minimum inhibitory concentration)-1 on PTP1B, a phosphate enzyme involved in various cellular processes. The optical density measurement at 620 nm provides a quantitative assessment of the inhibition of PTP1B activity by MIC-1 at different concentrations. The procedure of this assay describes the methodology used to assess the *in vitro* inhibitory activity of MIC-1 against PTP1B using a PTP1B inhibitor screening assay kit from Abcam. All experiments were performed as per the manufacturer’s protocol.

##### 2.4.1.1 Phosphate standard curve

Different volumes of PTP1B assay buffer and phosphate standard solutions were added to each well to generate a phosphate standard curve. The volumes used for the PTP1B assay buffer were 100, 97.5, 95, 90, 80, and 70 µL. The volumes used for 100 µM phosphate standard solutions were 0, 2.5, 5, 10, 20, and 30 µL.

##### 2.4.1.2 Preparation of MIC-1 and positive control suramin

Different concentrations of MIC-1 (10, 20, 40, 80, 160, or 320 µM) and the positive control suramin (1, 5, 10, 20, 40, or 80 µM) were prepared. For each concentration, 10 µL of compound was mixed with 35 µL of PTP1B assay buffer and 5 µL of PTP1B diluent, making a total reaction volume of 50 µL.

##### 2.4.1.3 Control samples

A “time zero” sample was prepared using 10 µL of dimethyl sulfoxide (DMSO), 35 µL of PTP1B assay buffer, and 5 µL of PTP1B diluent. A “control well” sample was prepared using 10 µL of DMSO and 40 µL of PTP1B assay buffer.

##### 2.4.1.4 Incubation, reaction stop, and measurement

PTP1B substrate (50 µL) was added to each well, and the reaction was incubated at 37 °C for 30 min. After incubation, 25 µL of red assay reagent was added to terminate the reaction. After an additional 20 min, absorbance was measured at 620 nm using a microplate reader. A standard curve was used to convert the optical density (OD) to nmol PO_4_^2−^.

The enzyme inhibition activity was calculated by the following formula:

$$\%\text{ Activity}\;=\;\frac{\left[\text{Test}\;\text{sample}\;\left(\text{nmol}\;{\text{PO}}_{4}{}^{-2}\right)-‘\text{time}\;\text{zero}’\;\left(\text{nmol}\;{\text{PO}}_{4}{}^{-2}\right)\right]}{\left[\text{Control}\;\left(\text{nmol}\;{\text{PO}}_{4}{}^{-2}\right)-‘\text{time}\;\text{zero}’\;\left(\text{nmol}\;{\text{PO}}_{4}{}^{-2}\right)\right]}\times 100$$

The PTP1B inhibition assay was conducted in three independent experiments in which different concentrations of each test compound were analyzed in triplicate (n = 3). The data was represented in terms of ± standard deviation (SD). Enzyme activity was analyzed by a spectrophotometer, and absorbance values were normalized with respect to the vehicle-treated control group showing 100% activity of PTP1B enzyme. The data given were assessed by two-way ANOVA, and a difference of 0.05 in *p*-value (*p* < 0.05) was considered significant.

### 2.5 Animal experimentation

The ondansetron derivatives that showed highest affinity against PTP1B and serotonin receptor were selected for further investigation. An animal model was established using streptozotocin (STZ) to induce diabetes. Male albino rats having an average weight of 140 g were taken from the local breeding facility at the Riphah Institute of Pharmaceutical Sciences, Riphah International University, Islamabad. The standard protocol was followed in which the rats were kept at standard room temperature of 18–22 °C. During this study, all rats were provided with a regular diet without any restriction on water consumption. All protocols followed in this study were recommended and approved by the Riphah Institute of Pharmaceutical Sciences (RIPS), Research and Ethical Committee (Approval ID: Ref. No.: REC/RIPS/2021/005).

### 2.6 Study design

Before introducing the rats to the laboratory settings, they were kept fasted for 10–12 h. Diabetes was introduced to the animals by injecting STZ intraperitoneally (i.p), and they were fed a high-fat diet. A fresh solution of STZ and 0.1 M citrate buffer at a pH of 4.5 was prepared and injected at a dose of 50 mg/kg. After 72 h of injecting STZ, the induction of diabetes was evaluated via tail prick. Rats having a blood glucose level of 280 mg/dL or more were considered to have developed diabetes (Akpojotor and Ebomoyi, 2021). The rats were divided into six groups (n = 4). Doses of saline (10 mL/kg) and STZ (50 mg/kg) were introduced intraperitoneally in groups I and II, respectively. Rats in the treatment groups (III, IV, and V), respectively, received 10 mg/kg doses of ondansetron, FN-10, and FN-06 *via* the intraperitoneal route, along with previously administered 50 mg/kg STZ. A dose of 500 mg/kg metformin was administered to group VI—the positive control group of the study. Blood glucose levels were monitored on days 1, 6, 12, 18, and 24 using a glucometer (Accu Chek active, Roche Pakistan Ltd).

### 2.7 Glycosylated hemoglobin test (HbA1c)

After 6 weeks, all six groups were subjected to HbA1c tests. A heart puncture procedure was employed to collect the blood samples (Doeing et al., 2003; Hou et al., 2022). The HbA1c test was performed at the National Lab and Diagnostics, Islamabad, Pakistan.

### 2.8 Oral glucose tolerance test (OGTT)

After fasting for 18 h, the rats were divided into six groups with four to five in each group. Doses of saline (10 mL/kg) and STZ (50 mg/kg) were introduced intraperitoneally in groups I and II, respectively. Rats in treatment groups III, IV, and V, respectively, received 10 mg/kg of ondansetron, FN-10, and FN-06 intraperitoneally , along with previously administered 50 mg/kg STZ. A dose of 500 mg/kg metformin was administered to group VI (positive control). All the treated groups were given an oral dose of 3 g/kg D-glucose. At different intervals (0, 30, 60, 90, and 120 min), blood glucose levels were monitored using an Accu Check Active glucometer (Roche Pakistan Ltd) (Ahmed et al., 2020).

### 2.9 Tissue visualization using hematoxylin and eosin staining

The liver and pancreas tissues were paraffinized and then deparaffinized in 100% xylene solution. Next, absolute ethanol was used to rehydrate the slides, after which dilutions of 70%–100% ethanol were used to rinse the slides, which were then rinsed with distill water. Before the slides were immersed into the hematoxylin for approximately 10 minutes, they were rinsed with phosphate buffer solution (PBS). Thence, all the slides were immersed for 5 min in water. A microscope was used to confirm their nuclear staining. Hematoxylin retreatment was performed if some portion of the slide was left untreated. The slides were then subject to treatment with 1% HCl mixed ammonia–water solution for approximately 5 min and eventually washed with water. The slides were subjected to eosin staining for approximately 5–10 min before they were washed with water and dried. Various ethanol dilutions (70%, 95%, and 100%) were used to dehydrate the slides, accompanied by xylene fixing and application of a cover slip. Microscopic images were observed using an Olympus light microscope (Olympus Japan). For further, detailed investigation pertaining to the shape of the tissue, five cellular infiltration and vacuole development pictures from each group were selected and analyzed using ImageJ software (Noman et al., 2022).

### 2.10 Analysis of liver enzymes

Cardiac puncture techniques were employed to collect blood samples from all groups. Blood samples were sent to the National Lab and Diagnostics, Islamabad, Pakistan to evaluation liver enzymes, including the levels of ALT (IU/L) (alanine transaminase), AST (IU/L) (aspartate aminotransferase), and TB (mg/dL) total bilirubin.

### 2.11 Statistical analysis

The given data were assessed by two-way ANOVA, followed by a *post hoc* Tuckey’s test. The results were shown in terms of mean ± standard error of the mean (SEM). A difference of 0.05 in the *p*-values (*p* < 0.05) was considered significant. GraphPad Prism (version 8.0.2) was employed for statistical analysis.

## 3 Results

### 3.1 Synthetic chemistry

**FIGURE 2:**
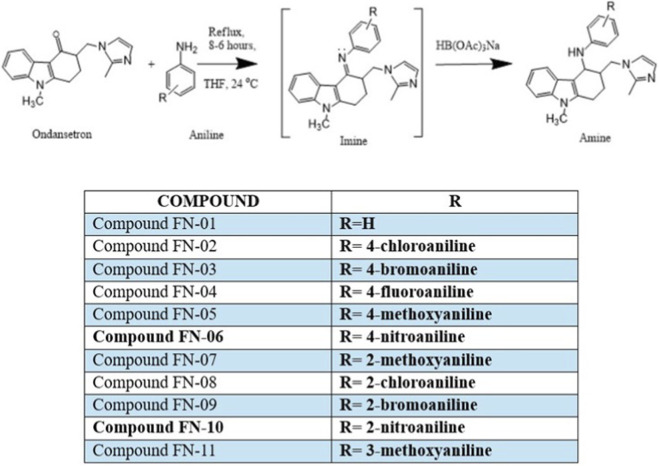
Chemical scheme for the synthesis of ondansetron derivatives (FN-01 to FN-11).

In total, 11 compounds were synthesized by developing a series of ondansetron derivatives by reductive amination reactions at the position 4 of the carbazole nucleus using tetrahydrofuran (THF) as a solvent at room temperature. Equimolar quantities of a ketone (ondansetron) and substituted aniline (amine) were refluxed for 6–8 h, with 1.3–1.6-g equivalent of sodium triacetoxyborohydride used as a reducing agent. A few drops of acetic acid were added to the reaction as a catalytic agent (Abdel-Magid et al., 1996). Monosubstituted anilines along with ondansetron were used as reactants to produce the respective products presented in Figure 2.

Compounds FN-06 and FN-10 were selected based on their computational data (docking score) for further analysis that included MD simulation and *in vivo* and *in vitro* studies. The progress of the reaction was determined by thin layer chromatography (TLC). On the TLC plate, the disappearance of the limiting reactant spot indicated the completion of the reaction. Stirring was stopped, and ice was added to the reaction mixture present in the round bottom flask to quench the reaction. Work-up was carried out by treating the organic layer (product) with DCM (dichloromethane) in a separating funnel and washing it two to three times with brine. The final product was dried using a rotary evaporator. The synthesized novel compounds were characterized by FT-IR and ^1^H-NMR.

### 3.2 FT-IR and ^1^H-NMR data

#### 3.2.1 Synthesis of FN-01

FT-IR (υ cm-1): 3379 (N-H stretching), 2983 (sp2 C-H), 2860 (sp3 C-H), 1640 (C=C), 1608 (C=N), 1399 (N-H bending), and 1281 (C-N). 1H NMR: (DMSO-d6, δ ppm) 9.93 (s, 1H, -NH), 7.69 (d, J=4Hz, 1H, imidazole-H), 7.58 (d, J=4Hz, 1H, imidazole-H), 7.56 (m, 5H, Ar-H), 7.30 (m, 4H, Ar-H), 3.75 (s, 3H, N-CH3), 3.61 (d, J=8Hz, 2H, CH2), 3.14 (s, 3H, Imidazole-CH3), 2.04 (m, 5H, cyclohexane), and 1.36 (d, J=4Hz, 1H, cyclohexane).

#### 3.2.2 Synthesis of FN-02

FT-IR (υ cm-1): 3320 (N-H stretching), 2965 (sp2 C-H), 2855 (sp3 C-H), 1620 (C=C), 1590 (C=N), 1369 (N-H bending), and 1294 (C-N). 1H NMR: (DMSO-d6, δ ppm) 10.08 (s, 1H, -NH), 8.29 (d, J=4Hz, 1H, imidazole-H), 7.63 (d, J=4Hz, 1H, imidazole-H), 7.60 (m, 4H, Ar-H), 7.33 (m, 4H, Ar-H), 3.75 (s, 3H, N-CH3), 2.60 (d, J=8Hz, 2H, CH2), 1.92 (s, 3H, Imidazole-CH3), 2.04 (m, 5H, cyclohexane), and 1.24 (d, J=4Hz, 1H, cyclohexane).

#### 3.2.3 Synthesis of FN-03

FT-IR (υ cm-1): 3343 (N-H stretching), 2954(sp2 C-H), 2843 (sp3 C-H), 1605 (C=C), 1596 (C=N), and 1375 (N-H bending), 1272 (C-N). 1H NMR: (DMSO-d6, δ ppm) 10.07 (s, 1H, -NH), 7.56 (d, J=4Hz, 1H, imidazole-H), 7.54 (d, J=4Hz, 1H, imidazole-H), 7.49 (m, 4H, Ar-H), 7.45 (m, 4H, Ar-H), 3.76 (s, 3H, N-CH3), 3.62 (d, J=8Hz, 2H, CH2), 3.32 (s, 3H, imidazole-CH3), 2.18 (m, 5H, cyclohexane), and 1.78 (d, J=4Hz, 1H, cyclohexane).

#### 3.2.4 Synthesis of FN-04

FT-IR (υ cm-1): 3290 (N-H stretching), 2981 (sp2 C-H), 2860 (sp3 C-H), 1590 (C=C), 1513 (C=N), 1354 (N-H bending), and 1263 (C-N). 1H NMR: (DMSO-d6, δ ppm) 9.99 (s, 1H, -NH), 7.60 (d, J=4Hz, 1H, imidazole-H), 7.56 (d, J=4Hz, 1H, imidazole-H), 7.15 (m, 5H, Ar-H), 7.11 (m, 4H, Ar-H), 3.75 (s, 3H, N-CH3), 2.63 (d, J=8Hz, 2H, CH2), 2.09 (s, 3H, imidazole-CH3), 2.03 (m, 5H, cyclohexane), and 1.24 (d, J=4Hz, 1H, cyclohexane).

#### 3.2.5 Synthesis of FN-05

FT-IR (υ cm-1): 3256 (N-H stretching), 2861 (sp2 C-H), 2840 (sp3 C-H), 1606 (C=C), 1575 (C=N), 1395 (N-H bending), and 1244 (C-N). 1H NMR: (DMSO-d6, δ ppm) 9.77 (s, 1H, -NH), 8.01 (d, J=4Hz, 1H, imidazole-H), 7.99 (d, J=4Hz, 1H, imidazole-H), 7.60 (m, 5H, Ar-H), 7.45 (m, 4H, Ar-H), 3.75 (s, 3H, N-CH3), 3.71 (d, J=8Hz, 2H, CH2), 3.15 (s, 3H, imidazole-CH3), 2.64 (d, J=8.0Hz, 2H) 2.09 (m, 5H, cyclohexane), and 1.35 (d, J=4Hz, 1H, cyclohexane).

#### 3.2.6 Synthesis of FN-06

FT-IR (υ cm-1): 3347 (N-H stretching), 2970 (sp2 C-H), 2807 (sp3 C-H), 1595 (C=C), 1528 (C=N), 1371 (N-H bending), and 1271 (C-N). 1H NMR: (DMSO-d6, δ ppm) 11.98 (s, 1H, -NH), 8.04 (d, J=4Hz, 1H, imidazole-H), 7.99 (d, J=4Hz, 1H, imidazole-H), 7.57 (m, 4H, Ar-H), 7.34 (m, 4H, Ar-H), 3.74 (s, 3H, N-CH3), 3.61 (d, J=8Hz, 2H, CH2), 3.15 (s, 3H, imidazole-CH3), 2.09 (m, 5H, cyclohexane), and 1.20 (d, J=4Hz, 1H, cyclohexane).

#### 3.2.7 Synthesis of FN-07

FT-IR (υ cm-1): 3305 (N-H stretching), 2913 (sp2 C-H), 2872 (sp3 C-H), 1607 (C=C), 1583 (C=N), 1353 (N-H bending), and 1210 (C-N). 1H NMR: (DMSO-d6, δ ppm) 8.04 (s, 1H, -NH), 7.56 (d, J=4Hz, 1H, imidazole-H), 7.24 (d, J=4Hz, 1H, imidazole-H), 7.03 (m, 5H, Ar-H), 6.81 (m, 4H, Ar-H), 3.75 (s, 3H, N-CH3), 3.60 (d, J=8Hz, 2H, CH2), 3.11 (s, 3H, imidazole-CH3), 2.08 (m, 5H, cyclohexane), and 1.36 (d, J=4Hz, 1H, cyclohexane).

#### 3.2.8 Synthesis of FN-08

FT-IR (υ cm-1): 3200 (N-H stretching), 2966 (sp2 C-H), 2884 (sp3 C-H), 1653 (C=C), 1540 (C=N), 1322 (N-H bending), and 1267 (C-N). 1H NMR: (DMSO-d6, δ ppm) 11.97 (s, 1H, -NH), 8.04 (d, J=4Hz, 1H, imidazole-H), 7.57 (d, J=4Hz, 1H, imidazole-H), 7.34 (m, 5H, Ar-H), 7.03 (m, 4H, Ar-H), 3.74 (s, 3H, N-CH3), 3.61 (d, J=8Hz, 2H, CH2), 3.14 (s, 3H, imidazole-CH3), 2.09 (m, 5H, cyclohexane), and 1.36 (d, J=4Hz, 1H, cyclohexane).

#### 3.2.9 Synthesis of FN-09

FT-IR (υ cm-1): 3260 (N-H stretching), 2940 (sp2 C-H), 2861 (sp3 C-H), 1618 (C=C), 1591 (C=N), 1370 (N-H bending), and 1242 (C-N). 1H NMR: (DMSO-d6, δ ppm) 11.98 (s, 1H, -NH), 8.04 (d, J=4Hz, 1H, imidazole-H), 7.57 (d, J=4Hz, 1H, imidazole-H), 7.34 (m, 5H, Ar-H), 7.03 (m, 4H, Ar-H), 3.73 (s, 3H, N-CH3), 3.61 (d, J=8Hz, 2H, CH2), 3.14 (s, 3H, imidazole-CH3), 2.19 (m, 5H, cyclohexane), and 1.36 (d, J=4Hz, 1H, cyclohexane).

#### 3.2.10 Synthesis of FN-10

FT-IR (υ cm-1): 3356 (N-H stretching), 2920 (sp2 C-H), 2853 (sp3 C-H), 1628 (C=C), 1584 (C=N), 1466 (N-H bending), and 1293 (C-N). 1H NMR: (DMSO-d6, δ ppm) 8.08 (s, 1H, -NH), 7.97 (d, J=4Hz, 1H, imidazole-H), 7.56 (d, J=4Hz, 1H, imidazole-H), 7.37 (m, 5H, Ar-H), 7.04 (m, 4H, Ar-H), 3.73 (s, 3H, N-CH3), 3.60 (d, J=8Hz, 2H, CH2), 3.32 (s, 3H, imidazole-CH_3_), 2.09 (m, 5H, cyclohexane), and 1.36 (d, J=4Hz, 1H, cyclohexane).

#### 3.2.11 Synthesis of FN-11

FT-IR (υ cm-1): 3367 (N-H stretching), 2931 (sp2 C-H), 2874 (sp3 C-H), 1642 (C=C), 1570 (C=N), 1427 (N-H bending), and 1265 (C-N). 1H NMR: (DMSO-d6, δ ppm) 9.96 (s, 1H, -NH), 8.01 (d, J=4Hz, 1H, imidazole-H), 7.56 (d, J=4Hz, 1H, imidazole-H), 7.36 (m, 5H, Ar-H), 7.05 (m, 4H, Ar-H), 3.75 (s, 3H, N-CH3), 3.61 (d, J=8Hz, 2H, CH2), 3.14 (s, 3H, imidazole-CH3), 2.03 (m, 5H, cyclohexane), and 1.36 (d, J=4Hz, 1H, cyclohexane).

### 3.3 Scheme of synthesis

#### 3.3.1 Molecular docking analysis

**FIGURE 3:**
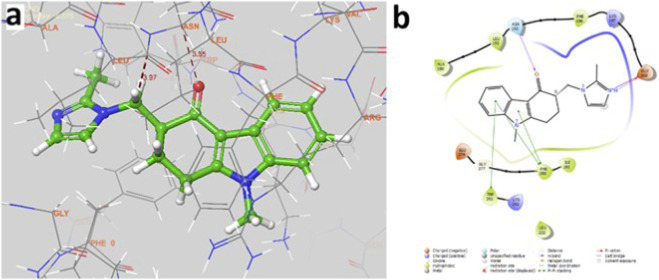
Three dimensional **(a)** and two dimensional **(b)** docked images of ondansetron against target enzyme protein tyrosine phosphatase 1B (PTP1B) having PDB ID (1T48).

**FIGURE 4:**
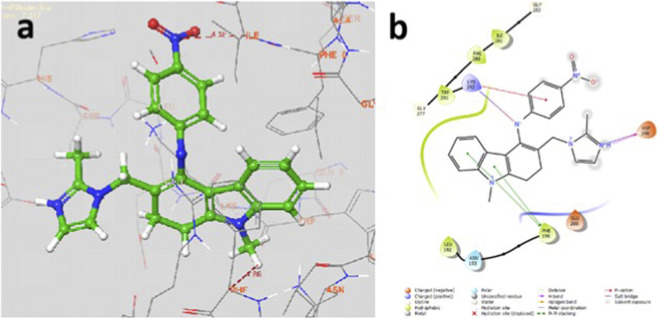
Three dimensional **(a)** and two dimensional **(b)** docked images of FN-06 against target enzyme protein tyrosine phosphatase 1B (PTP1B) having PDB ID (1T48).

**FIGURE 5:**
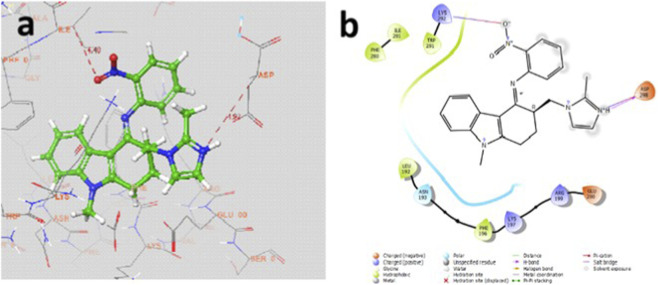
Three dimensional **(a)** and two dimensional **(b)** docked images of FN-10 against target enzyme protein tyrosine phosphatase 1B (PTP1B) having PDB ID (1T48).

Molecular docking studies of the synthesized derivatives were conducted against PTP1B (PDB ID: 1T48) and 5-HT_3_ (PDB ID: 6W1M). Interestingly, ondansetron (parent compound) showed the highest binding affinity against both the pockets PTP1B and 5-HT_3_, revealing docking score values of −8.331 kcal/mol and −11.76 kcal/mol, respectively (Figure 3). Against the PTP1B pocket, the highest binding affinity was by compound FN-06 with a docking score of −7.317 kcal/mol (Figure 4), and the second highest binding affinity against the PTP1B receptor was shown by compound FN-10 with a docking score of −6.291 kcal/mol (Figure 5). Halogens such as chlorine (Cl), bromine (Br), and fluorine (F) along with polar functional groups such as methoxy (OCH_3_), when attached to the phenyl ring of the ondansetron derivatives, were inferior in developing binding associations with the protein PTP1B compared to the nitro group (NO_2_), which had a superior binding ability. It is worth noting that compounds substituted with the nitro (NO_2_) functional group showed highest binding affinity against both PTP1B and 5-HT_3_ receptors. As shown from the results in Table 1, drug candidates (ondansetron, FN-10, and FN-06), which showed strong binding affinities with 5-HT_3_ receptors, also tend to bind with the PTP1B receptor with a reasonably adequate binding strength.

**TABLE 1:** Docking scores and binding interactions of top selected ligands FN-06 and FN-10 with target enzymes PTP1B and 5-HT3.

Protein	Compound (ligands)	Docking score (kcal/mol)	Glide energy (KJ/mol)	MMGBSA	Interacting residues
PTP1B (1T48)	Ondansetron	−8.331	−36.959	−44.48	H-Bond: ASN 193, GLU 200
​	​	​	​	​	Hydrophobic interactions: ALA 189, LEU 192, PHE 196, TRP 291, PHE 280, ILE 281, LEU 232
​	FN-06	−7.317	−40.699	−31.89	H-Bond: ASP 298
​	​	​	​	​	Hydrophobic interactions: TRP 291, PHE 280, ILE 281, PHE 196, LEU 192
​	FN-10	−6.291	−41.228	-	H-Bond: ASP 298
​	​	​	​	​	Hydrophobic interactions: PHE 196, LEU 192, PHE 180, ILE 281, TRP 291
5-HT_3_ (6W1M)	Ondansetron	−11.768	−50.189	−85.78	H-Bond: -
​	​	​	​	​	Hydrophobic interactions: ILE 44, VAL 43, TYR 126, TYR 64, TRP 63, TYR 207, TRP 156, PHE 199, ILE 201
​	FN-10	−11.598	−53.788	−71.27	H-bond: TRP 156
​	​	​	​	​	Hydrophobic interactions: ILE 44, VAL 43, TYR 64, TRP 63, TYR 126, TYR 207, LEU 157
​	FN-06	−10.903	−42.475	−52.67	H-bond: TRP 156
​	​	​	​	​	Hydrophobic interactions: ILE 180, TYR 126, VAL 43, ILE 44, TYR 64, TRP 63, TYR 207, PHE 199, ILE 201

### 3.4 Molecular dynamic simulation

#### 3.4.1 RMSD analysis

**FIGURE 6:**
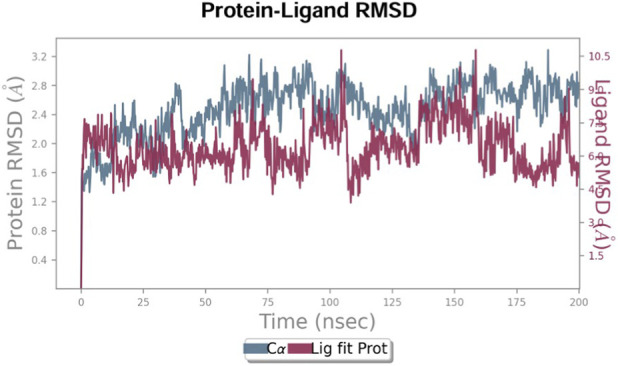
RMSD plot of the docked complex of ligand FN-06 with protein PTP1B. The average RMSD calculations observed for FN-06 are represented in red and the average RMSD calculations observed for protein PTP1B are represented in blue.

The MD simulation study was performed at 200 ns, at which the fluctuations in the protein–ligand complex were determined by observing the root mean square deviation (RMSD) plot (Figure 6). The main purpose of performing this study was to identify the flexibility of the protein, the average distance between the atoms of protein, and equilibration. After performing the molecular dynamic simulation process, it was revealed that ligand FN-06 showed stable binding through amino acids GLN 123 and ARG 199. These were considered crucial for the stable binding of FN-06 with the protein’s (PTP1B) pocket. Other residues such as ASN 90 also contributed to establishing stable connections to the target but to a lesser extent.

In this case, the protein–ligand complex showed reasonable stability to 100 ns with some fluctuations that progressed to 200 ns. The average RMSD calculated for protein (PTP1B) was 2.48 ± 0.36 Å, as represented in blue in Figure 6. Some minor fluctuations were observed up to 100 ns, which diminished as the simulation continued to 200 ns. The average RMSD calculated for ligand FN-06 was 6.39 ± 1.02 Å (in red). In terms of values, the RMSD results were encouraging, but notable fluctuations were observed during the simulation. These results are indicative of modifications in the structure of the ligand, which may improve overall stability in the ligand–protein complex interactions.

#### 3.4.2 RMSF analysis

**FIGURE 7:**
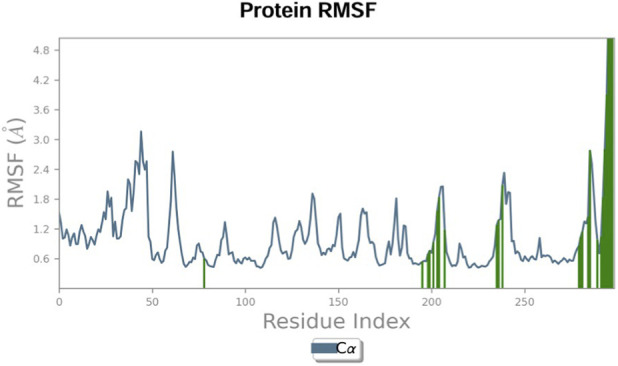
RMSF plot of the docked complex of ligand FN-06 with protein PTP1B exhibiting overall fluctuations of the amino acid residues of the protein after ligand binding.

**FIGURE 8:**
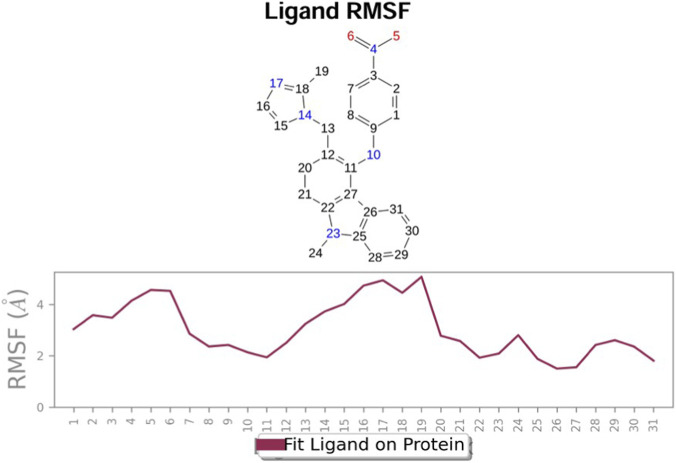
RMSF plot of the docked complex of ligand FN-06 with protein PTP1B.

The fluctuations in the overall protein structure were calculated by the root mean square fluctuation (RMSF) plot, thereby indicating the flexible region of the protein (Figure 7). In this method, the average movement of atoms at a particular temperature and pressure is measured. The lower the values of RMSF plot, the greater the stability, whereas higher RMSF values express lesser stability. RMSF analysis was carried out to monitor the fluctuations in the residues of the protein–ligand (PTP1B–FN-06) complex. It also included the analysis of flexibility of the PTP1B and FN-06 residues. The RMSF plot in Figures 7, 8 fulfils the criteria of a stable complex.

#### 3.4.3 Radius of gyration

**FIGURE 9:**
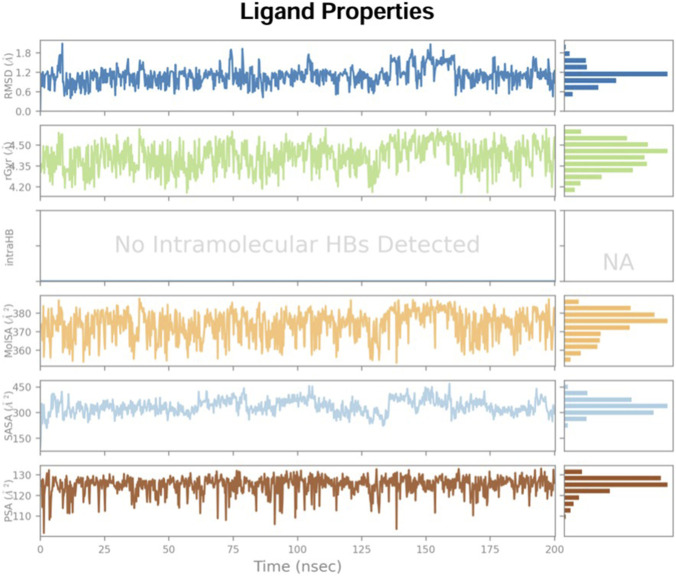
Plots of the docked complex of ligand FN-06 with protein PTP1B exhibiting interaction between the protein–ligand complex and the solvents.

This parameter is fundamental in assessing the stable folding and unfolding (extendedness) of the protein–ligand complex during the simulation. It also indicates the variation in the compactness of this complex. The value of the radius of gyration (Rg) is stable, exhibiting the adequate compactness of the protein–ligand complex. A protein that maintains a relatively consistent value indicates that it has folded in a stable manner; on the other hand, variation in the Rg value over a specific time shows the unfolding of the protein (Ghasemi et al., 2016). Consistent values of Rg are observed in Figure 9, indicating its compactness and stability.

#### 3.4.4 Solvent accessible surface area

The solvent accessible surface area (SASA) is used to measure the interactions between the solvents and the protein–ligand complex. This parameter helps predict the conformational changes during the protein–ligand interaction. The SASA plot is displayed in Figure 9 and indicates that the average SASA value is 335.18 ± 41.82 Å^2^ and the peak appears at approximately 460 Å^2^. These results indicate that the protein–ligand complex is stable without showing alteration in the structure.

#### 3.4.5 ADMET profile

Out of the 11 synthesized compounds (ondansetron derivatives), the top two ligands—FN-06 and FN-10—showing the maximum affinity for the proteins PTP1B and 5 HT3 were analyzed for their pharmacokinetic profiles (Table 2). Both these compounds tend to obey the Lipinski rule with no violations (Table 2). Their molecular weight was found to be less than 500 Da, and they showed high GI absorption. Moreover, these compounds also account for the property of druggability as a moderate to good bioavailability score was exhibited by these compounds that will aid in making the drug discovery process less challenging (Pantaleão et al., 2022). Unlike ondansetron, its derivatives FN-06 and FN-10 were impermeable to the blood–brain barrier, which indicates their superior safety profile and reduced adverse effects associated with CNS. The synthetic accessibility of compounds FN-06 and FN-10 lies at the moderate range, varying from 4.12 to 4.24, respectively.

**TABLE 2:** Physicochemical properties of ondansetron and its top selected derivatives (FN-10 and FN-06).

Properties	Ondansetron	FN-06	FN-10
Molecular formula	C_18_H_19_N_3_O	C_24_H_25_N_5_O_2_	C_24_H_25_N_5_O_2_
Molecular weight	293.36 g/mol	415.49 g/mol	415.49 g/mol
logP (polarizability)	2.57	2.89	3.13
log Kp (skin permeability)	−6.26 cm/s	−5.89 cm/s	−5.50 cm/s
H-bond donors	0	1	1
H-bond acceptors	2	3	3
BBB (permeability)	Yes	No	No
GI absorption	High	High	High
Molar refractivity	87.39	124.62	124.62
TPSA	39.82 A^2^	80.60 A^2^	80.60 A^2^

### 3.5 *In vitro* analysis

#### 3.5.1 Protein tyrosine phosphatase 1B inhibition assay

The inhibitory activity of the nPTP1B enzyme was evaluated using a PTP1B inhibitor Screening Assay Kit (ab 139465, Abcam).

**FIGURE 10:**
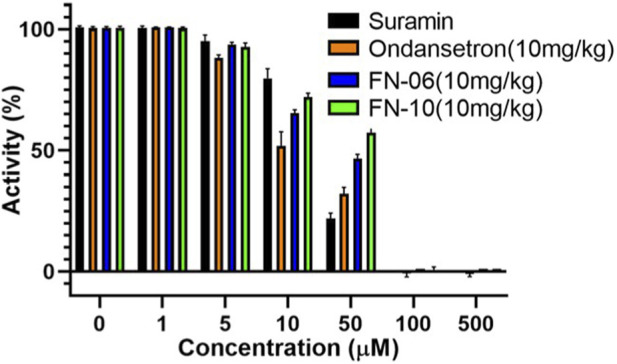
Concentration-dependent inhibition of enzyme PTP1B by ondansetron, FN-06, FN-10, and suramin (standard).

The activity of various test groups is presented in Figure 10. With suramin as the positive control, ondansetron and its synthesized derivatives were tested at concentrations of 0, 1, 5, 10, 50, 100, and 500 µM, with the result represented in percentage activity. Suramin showed nearly 100% activity at lower concentrations, with a similar trend represented by ondansetron. The effects of FN-06 and FN-10 were similar, with a similar concentration-dependent pattern. Of the two synthesized compounds, FN-10 showed stronger activity, particularly in mid-range concentrations such as 5–50 µM. The IC_50_ values for suramin, ondansetron (10 mg/kg), FN-06 (10 mg/kg), and FN-10 (10 mg/kg) are 25.84, 17.11, 30.49, and 42.66, respectively.

#### 3.5.2 *In vivo* anti-diabetic analysis

**FIGURE 11:**
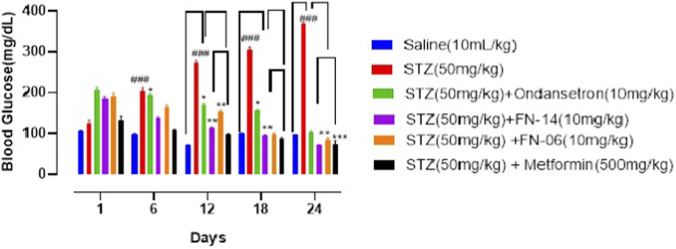
Effect of ondansetron derivatives on blood glucose levels in animal models for 24 days. Data obtained are presented as the mean ± SEM (*n* = 4–5). Two-way ANOVA, followed by a *post hoc* Tukey’s test, was used. *p*-values are presented as ^###^ *p* < 0.001 vs. saline and **p < 0.01 and ***p < 0.001 vs. STZ-treated mice.

Ondansetron and its derivatives were tested in a type 2 diabetes model based on male albino rats. A significant decrease in the blood glucose levels of the test animals was observed (Figure 11). Metformin was taken as the positive control, while only the compounds FN-06 and FN-10 showing the highest binding affinity in computational studies were selected for animal experimentation.

An elevated lipid profile is usually associated with diabetic patients along with erratic blood glucose levels. The role of brucine in these conditions is commendable as it is beneficial in addressing both these issues. As brucine has the capacity to manage both hyperglycemia and obesity, this makes it unique compared to other treatment options such as metformin, which only has the ability to reduce increased blood glucose levels. The management of blood sugar levels and the lowering of body weight makes brucine a promising candidate for a therapeutic agent for conditions such as diabetes and obesity. However, brucine’s toxic profile decreased its popularity, and agents such as ondansetron along with its derivatives can be used for anti-diabetic action after proper screening and clinical trials.

#### 3.5.3 Measurement of body weight and blood glucose levels

The rats were divided into six groups (I to VI), and diabetes was induced in each group apart from the saline group.

##### 3.5.3.1 DAY 1

The treatment groups (OND, FN-06, and FN-10) showed significant results on day 1 in comparison to the control and saline groups. In comparison, STZ vs group VI and FN-10 vs FN-06, with *p*-values 0.6217 and 0.8973, were simultaneously observed with no significant difference. However, significant differences were observed with OND along with FN-06 or FN-10 in comparison with the saline group, whereas relatively less difference was observed between FN-10 vs FN-06 (*p* = 0.8973). A significant difference (*p* = 0.0002) was also observed among the saline and other treatment groups, which may reveal separate results altogether.

##### 3.5.3.2 DAY 6

On day 6, significant results were observed for treatment groups FN-10 and FN-06. Significant reduction was observed in these groups showing a *p*-value < 0.0001. In comparing FN-10 and FN-06, the former produces a more prominent and strong positive effect in reducing blood glucose levels and body weight. On the other hand, no significant difference was observed in group VI and the saline group, but improved significant difference was observed when comparing group VI with the STZ and treatment groups. A prominent negative effect for the STZ group was observed with respect to the saline group. This negative impact of STZ improves when the rats are treated with FN-10 and FN-06.

##### 3.5.3.3 DAY 12

It is evident from the results that the diseased or STZ group showed adverse effects, demonstrating the negative role of STZ in this study. FN-10 and FN-06, when administered to the diseased group, lowered the blood glucose level, with the effect of FN-10 more pronounced than of FN-06. Group VI (STZ group treated with metformin) showed relatively better regular results with respect to other groups. The results of FN-10 resemble the outcome of group VI, suggesting better therapeutic action than FN-06. In general, FN-10 showed the most promising effects to counteract the negative effects of STZ.

##### 3.5.3.4 DAY 18

The negative effects of STZ in the diseased group continued to increase as the study progressed. Considering the efficacy of FN-10 and FN-06, no prominent difference was present between the two groups. Thus, the ability of FN-10 and FN-06 to produce the desired results was relative to the effectiveness of the diseased group treated with metformin. Animals treated with metformin had slightly better outcomes than those treated with ondansetron, indicating that the latter holds an intermediate position in the study.

##### 3.5.3.5 DAY 24

On day 24 of the study, significant differences were observed between the STZ group and other treatment groups. There was a significant difference between FN-10 and the saline group, but no significant difference between FN-10 and FN-06. Significant differences were observed between the groups treated with metformin and the saline group.

The treatment groups (OND, FN-10, and FN-06) produced significant and prominent results in comparison with the saline (p = 0.0002) and control groups (Figure 11). On the other hand, no significant difference appeared between FN-10 and FN-06, with respective *p*-values of 0.6217 and 0.8973. In these treatment groups (FN-10 and FN-06), significant reductions in body weight and blood glucose levels were observed on the sixth day of the study, with *p-*values less than 0.0001. It is evident from the results that STZ played a negative role in escalating the harmful effects, whereas FN-10 and FN-06 decreased blood glucose levels: the effect of FN-10 was more prominent. The action of FN-10 was comparable to the metformin group, thereby indicating it to be a more potent drug than FN-06. This statistical data indicates useful information about the effectiveness and results of these treatments.

### 3.6 Glycosylated hemoglobin test (HbA1c)

The animals (rats) present in all six groups were subjected to HbA1c tests after a 6 weeks, and very promising results were observed in the treatment groups (OND, FN-10, and FN-06), with minimum values as shown in Table 3.

**TABLE 3:** Results of concentration-dependent effects of ondansetron, FN-10, and FN-06 on HbA1C test in all six groups (I-VI) of the animal model.

Groups (I-VI)	HbA1C (%)
Group I/saline (10 mL/kg)	**4.2 ± 0.05**
Group II/STZ (50 mg/kg)	**9.95 ± 0.08**
Group III/STZ (50 mg/kg) + ondansetron (10 mg/kg)	**4.1 ± 0.46**
Group IV/STZ (50 mg/kg) + FN-10 (10 mg/kg)	**4.3 ± 0.11**
Group V/STZ (50 mg/kg) + FN-06 (10 mg/kg)	**3.9 ± 0.11**
Group VI/STZ (50 mg/kg) + metformin (500 mg/kg)	6.15 ± 0.02

### 3.7 Oral glucose tolerance test

**FIGURE 12:**
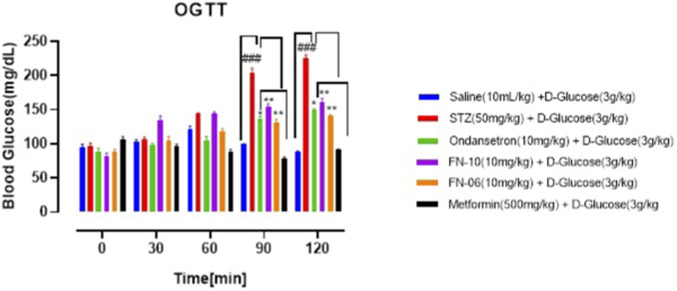
Effect of ondansetron derivatives on blood glucose levels in an animal model. Data obtained are presented as the mean ± SEM (*n* = 4–5). Two-way ANOVA, followed by a *post hoc* Tukey’s test, was used. *p*-values are presented as ^###^ *p* < 0.001 vs. saline and **p < 0.01 and ***p < 0.001 vs. STZ-treated mice.

After administering a dose of 3 g/kg D-glucose to all the groups, an oral glucose tolerance test was performed to observe the blood glucose levels at different time intervals. Thereafter, encouraging results were also recorded in the three treatment groups (OND, FN-10, and FN-06) (Figure 12).

### 3.8 Liver enzymes

The elevated levels of liver enzymes (ALT and AST) indicated the negative impact of STZ, thereby characterizing symptoms of liver injury. Different degrees of protective actions were observed in the treatment groups (ondansetron, FN-10, and FN-06) (Table 4). FN-10, being the most potent, showed prominent results as the levels of liver enzymes (ALT and AST) were reduced to normal. Ondansetron, on the other hand, offered partial liver protection in which the results of ALT were satisfactory but rather unsatisfactory for AST. The protection action of FN-06 was similar to that of FN-10 but slightly on the weaker side. Metformin, being the positive control of the study, offered normal and consistent values against liver injury induced by STZ. FN-10 showed a significant difference with respect to the positive control in normalizing AST values.

**TABLE 4:** Effect of ondansetron and its derivatives (FN-10 and FN-06) on liver enzymes.

Groups (I-VI)	AST (IU/L)	ALT (IU/L)	TB (mg/dL)
Group-I/Saline (10 mL/kg)	179.5 ± 63.79	54 ± 12.70	0.901 ± 0.004
Group-II/STZ (50 mg/kg)	339.5 ± 90.93	388 ± 181.2	2.885 ± 0.285
Group-III/STZ (50 mg/kg) + ondansetron (10 mg/kg)	209.5 ± 11.83	221.5 ± 21.65	1.302 ± 0.283
Group-IV/STZ (50 mg/kg) + FN-10 (10 mg/kg)	111.5 ± 33.19	136 ± 8.66	0.526 ± 0.060
Group-V/STZ (50 mg/kg) + FN-06 (10 mg/kg)	191 ± 8.082	144 ± 6.92	0.65 ± 0.063
Group-VI/STZ (50 mg/kg) + metformin (500 mg/kg)	154.5 ± 21.65	99.5 ± 1.44	0.8 ± 0.06

As mentioned earlier, the action of STZ suggests its negative role leading to liver injury, and it was involved in enhancing the levels of total bilirubin (TB). Limited protection against liver injury was provided by ondansetron, which resulted in some decrease in the total bilirubin levels but were above normal levels. Comprehensive protection was observed in the treatment groups (FN-10 and FN-06) and the positive control group (metformin), which resulted in a marked decrease in total bilirubin (TB) levels. Moderate liver protection was, therefore, observed in the group treated with ondansetron.

### 3.9 Hematoxylin and eosin staining

**FIGURE 13:**
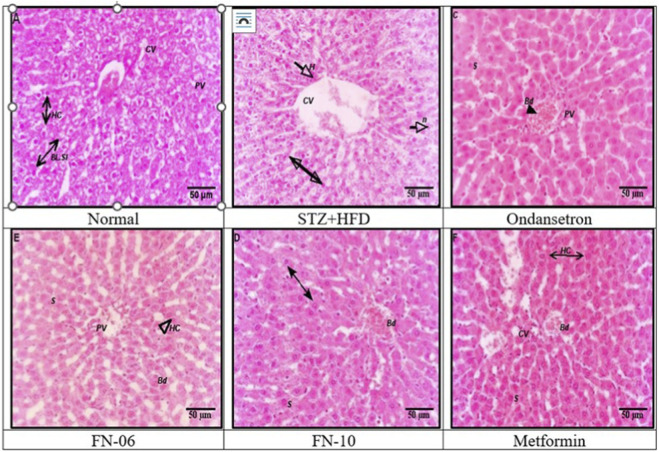
Histomorphological images of rat liver segments stained with H and E (40×). **(A)** Normal hepatocytes (HC), blood sinusoids (BL.SI), central veins (CV), and portal veins (PV) in the liver of the normal group. **(B)** The HFD/STZ diabetic liver group exhibits swelling of the hepatic cell **(H)**, pyknotic nuclei (n), and central vein (CV). The nuclei of some injured hepatocytes were lost (double-headed arrows). **(C)** Ondansetron group displays the portal region with the bile duct (Bd) and portal vein (PV) encircled by healthy hepatocytes and divided by neatly arranged blood sinusoids (S). **(D,E)** Normal portal vein (PV) with bile duct (Bd), normal hepatocyte (HC), and blood sinusoids present in the FN-10 and FN-06 treatment groups. **(F)** Somewhat normal structures with normal hepatocytes (HC), blood sinusoids (S), central vein (CV) with bile duct (Bd) in the metformin standard group. **(A–F)**: Scale bar 50 µm analyzed using ImageJ software. KEY: CV, central veins; PV, portal veins; HC, hepatocyte; Bd, bile duct.

Liver and pancreatic tissues of the saline group (10 mL/kg) showed normal architecture without any pathological alterations. The group of rats induced with STZ (50 mg/kg) and a high-fat diet showed distorted liver and pancreatic tissues along with abnormal architecture, deterioration of hepatocytes, and edema. These abnormal liver and pancreatic cells were restored with mild sinusoidal congestion in the animal groups treated with ondansetron (10 mg/kg), FN-10 (10 mg/kg), FN-06 (10 mg/kg), and metformin (Figure 13).

A significant difference was observed in the liver slides of the high-fat diet + STZ treated groups compared to the treatment groups comprising ondansetron (10 mg/kg), FN-10 (10 mg/kg), and FN-06 (10 mg/kg). Highly significant p values less than 0.0001 were observed, indicating a prominent difference between the high fat diet + STZ and other treatment groups. A strong hepatoprotective action of the treatment groups FN-10 and FN-06 was observed, whereas ondansetron showed relatively less effective action. Positive and consistent results were observed in the slides of the groups treated with metformin. Ondansetron had limited hepatoprotective activity compared to its derivatives FN-10 and FN-06.

**FIGURE 14:**
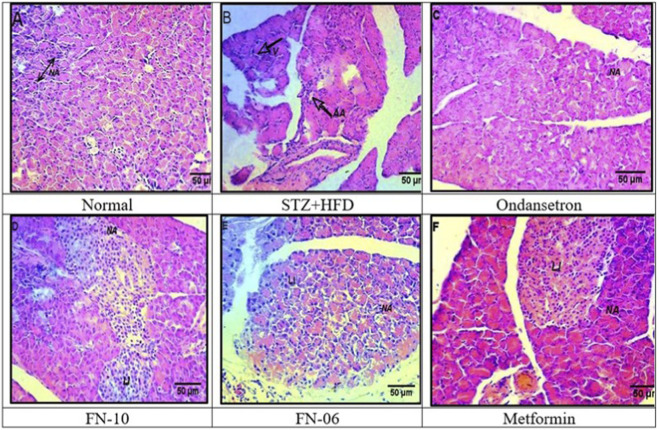
Rat pancreatic histopathological sections stained with H&E. **(A)** Normal control (normal islet cells in the pancreas). **(B)** Diabetic group experienced vacuolization and atrophy acini with infiltration pancreatic cell. **(C)** Ondansetron group received 10 mg/kg, showing reduction of dilated pancreatic cell with normal islets of Langerhans. **(D,E)** Both derivatives FN-06 and FN-10 received 10 mg/kg, showing normal pancreatic acini with islets of Langerhans. **(F)** Group received metformin (normal histology of islets of Langerhans). Scale bar: 50 µm using ImageJ software. Key: NA, normal pancreatic acini; V, vacuole; AA, atrophy acini; LI, islets of Langerhans.

The damaging effects on pancreatic tissues in the animal groups treated with high-fat diet + STZ were clear and prominent. FN-06 emerges as the most effective and promising derivative of ondansetron with respect to its therapeutic action on damaged pancreatic tissues. The action of FN-06 is more pronounced in an animal model, probably due to activation of complex processes that take place within a living body such as metabolic activation of drug molecules and their distribution, which were absent while performing *in vitro* studies. The more curative results of FN-06 compared to metformin, ondansetron, and FN-10 indicate its role as a comprehensive treatment option for damaged pancreatic tissues (Figure 14). Metformin was consistent in its performance but was less effective than FN-06. The therapeutic action of ondansetron and FN-10 was stronger than metformin but less potent than FN-06. When considering ondansetron along with its derivatives FN-06 and FN-10, F-06 in particular appears an excellent drug of choice for the treatment of compromised pancreatic cells.

## 4 Conclusion

We successfully synthesized the ondansetron derivatives FN-01 to FN-11 by following a simple reaction route with good to excellent yields as PTP1B inhibitors. Multiple attempts have been made in recent years to synthesize PTP1B inhibitors but, in most cases, complications were associated with the selectivity and toxicity of the synthesized analogs, which resulted in the failure of their clinical trials. The synthesized ondansetron derivatives were analyzed as PTP1B inhibitors to study the role of enzyme PTP1B in lowering blood glucose levels in conditions such as type 2 diabetes. Encouraging results were obtained when the synthesized PTP1B inhibitors were investigated by *in silico*, *in vitro*, and *in vivo* studies. Favorable *in silico* data of the selected ligand (FN-06) revealed maximum binding affinity with protein PTP1B (1T48), with a docking score of −7.317 kcal/mol, which was also observed in the results of the molecular dynamic study of this ligand. The selected ondansetron derivatives FN-06 and FN-10 proved to be instrumental in demonstrating the inhibition of enzyme PTP1B in case of both enzyme inhibition assay performed in the lab and in the animal model of the rats. Furthermore, it is evident from the outcomes of the study that FN-06 and FN-10 are effective in producing antidiabetic and hepatoprotective action by limiting the overexpression of enzyme PTP1B. The findings of this study further suggest that ondansetron and its derivatives may serve as lead structures to design potent drug molecules with antidiabetic and hepatoprotective activities. Pharmacological, toxicological, and clinical studies of the highly potent derivatives FN-06 and FN-10 may provide valuable insights into developing improved treatment options for diabetes.

## Data Availability

The datasets presented in this study can be found in online repositories. The names of the repository/repositories and accession number(s) can be found in the article/Supplementary Material.
