# Supplementary material for: Ondansetron derivatives as potential PTP1B inhibitors for treating type 2 diabetes mellitus: in silico, in vitro, and in vivo analysis
Source: Front Pharmacol. 2026 Jun 3;17:1796359. doi: 10.3389/fphar.2026.1796359 (PMC13272487; doi:10.3389/fphar.2026.1796359)
Supplement: Supplementary file 1 [file Supplementaryfile1.docx]

**Supplementary Information**

- 2D and 3D images of the synthesized compounds against PTP1B pocket.

| 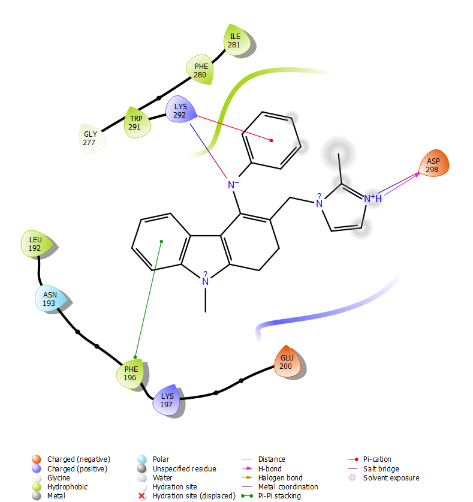 | 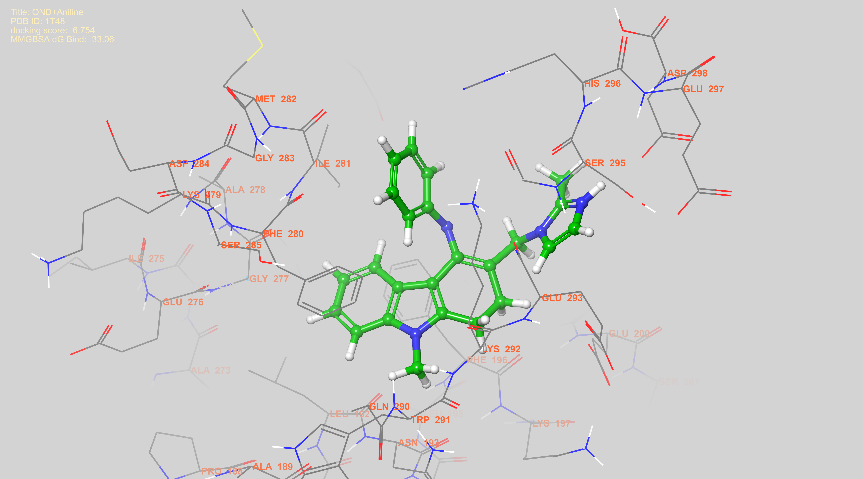 |
| --- | --- |
| 2D image & 3D images of FN-01 | |
| 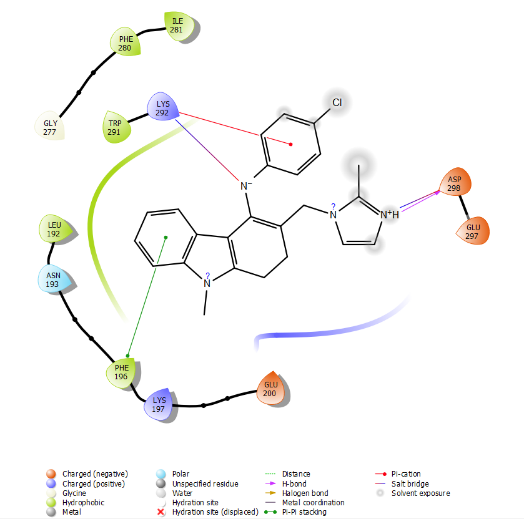 | 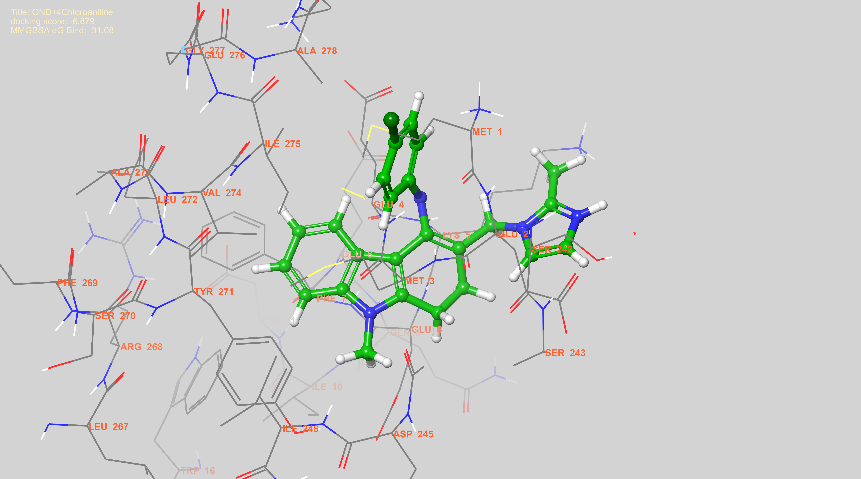 |
| 2D image & 3D images of FN-02 | |
| 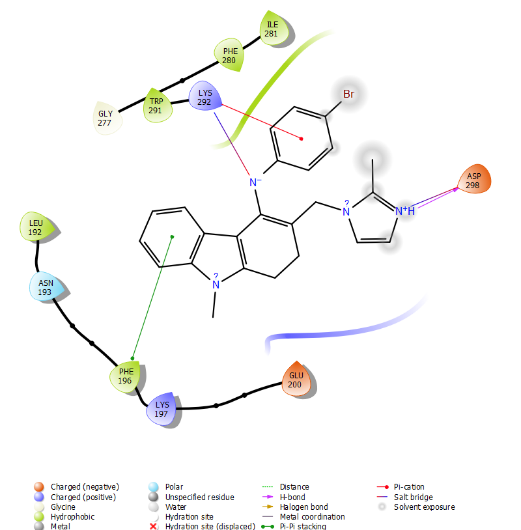 | 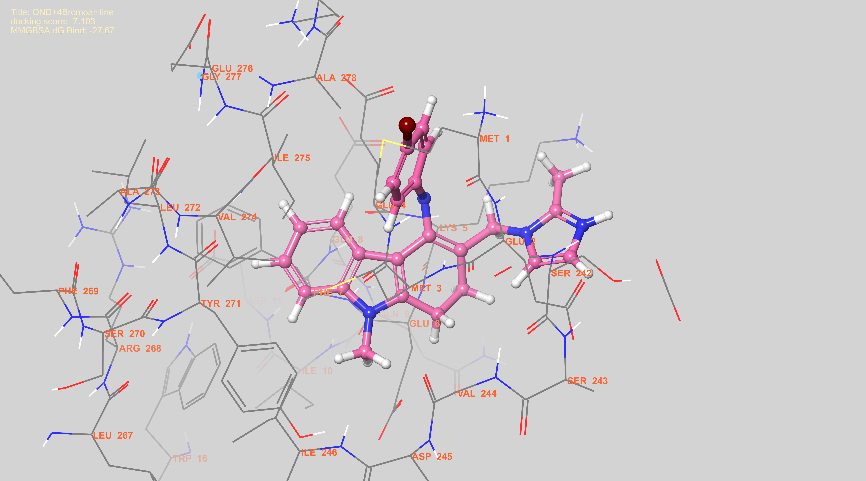 |
| 2D image & 3D images of FN-03 | |
| 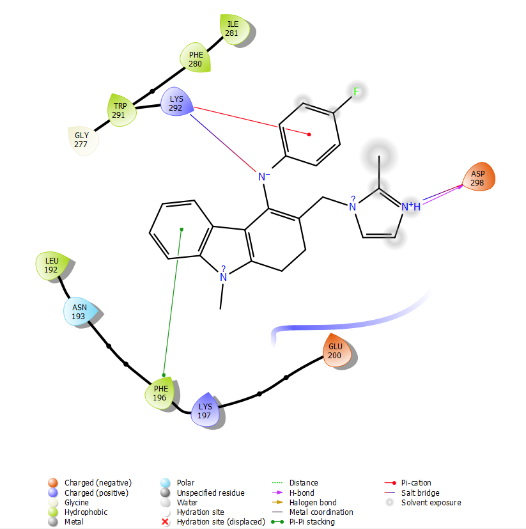 | 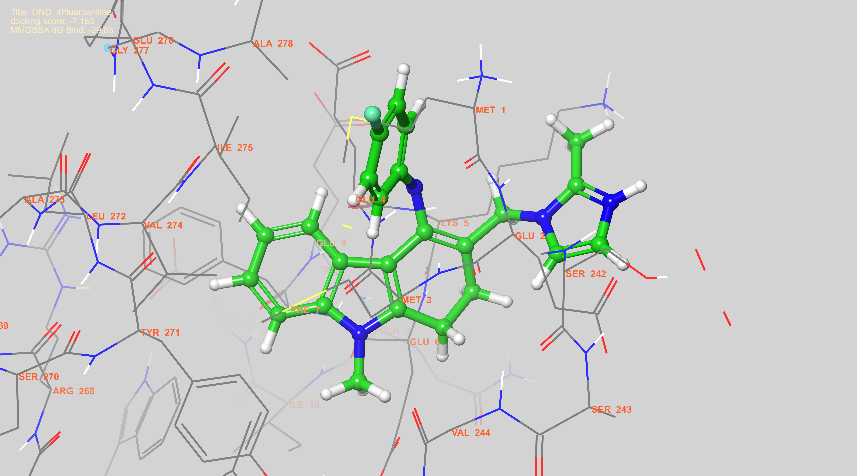 |
| 2D image & 3D images of FN-04 | |
| 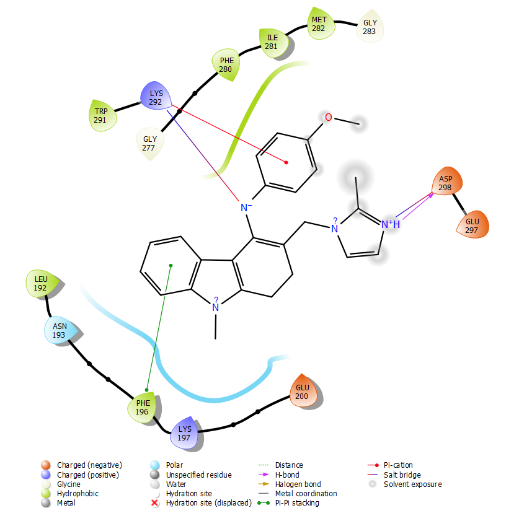 | 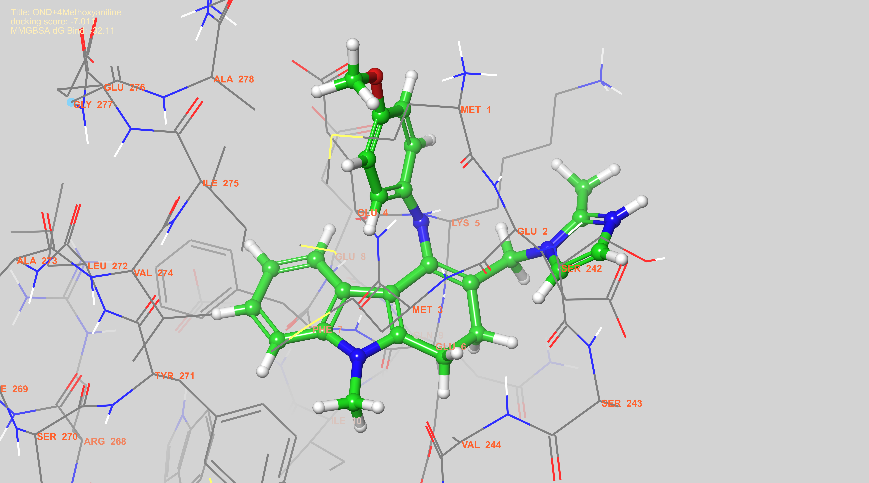 |
| 2D image & 3D images of FN-05 | |
| 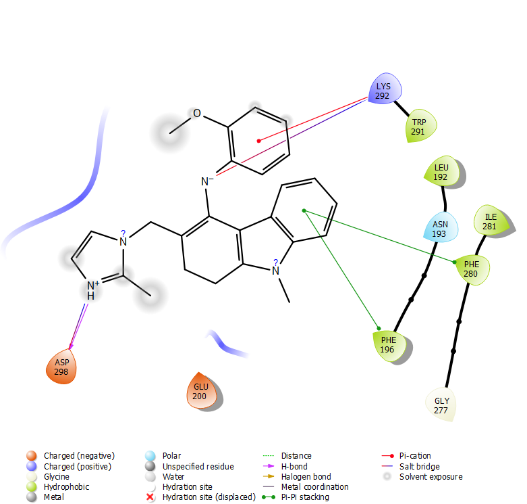 | 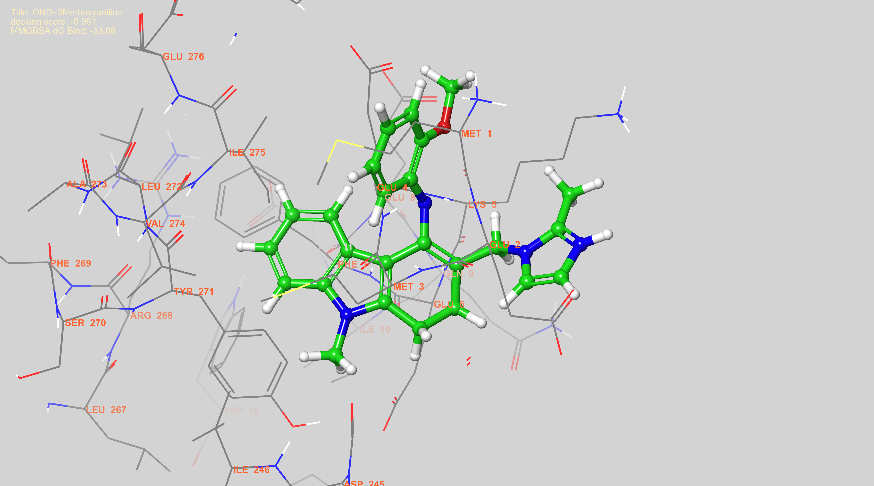 |
| 2D image & 3D images of FN-07 | |
| 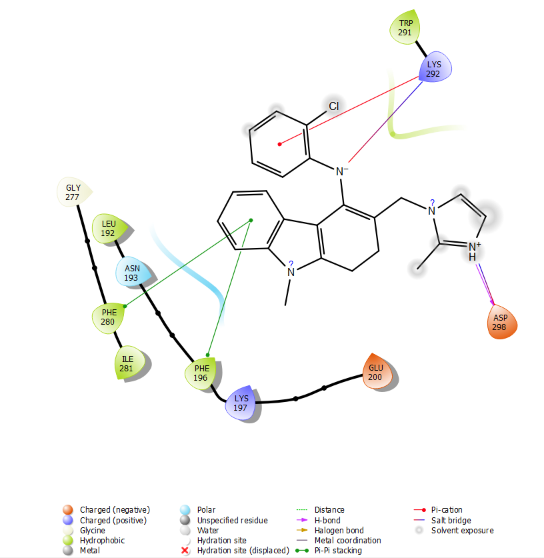 | 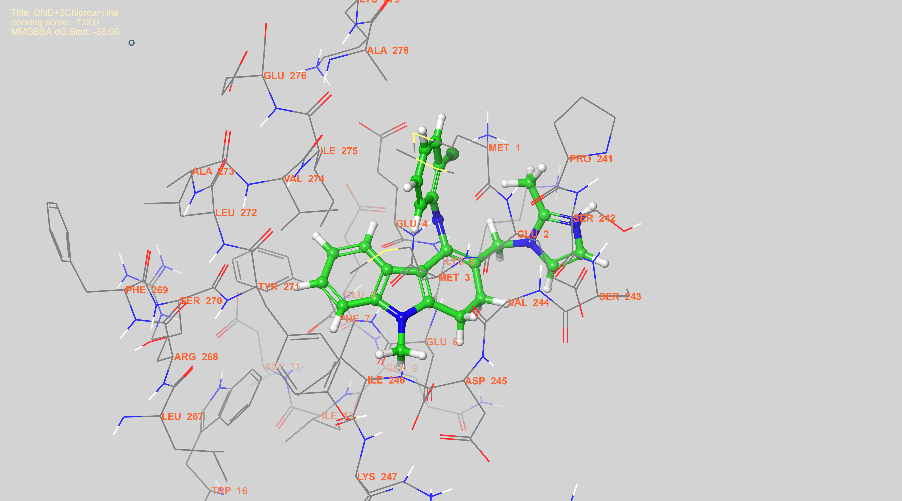 |
| 2D image & 3D images of FN-08 | |
| 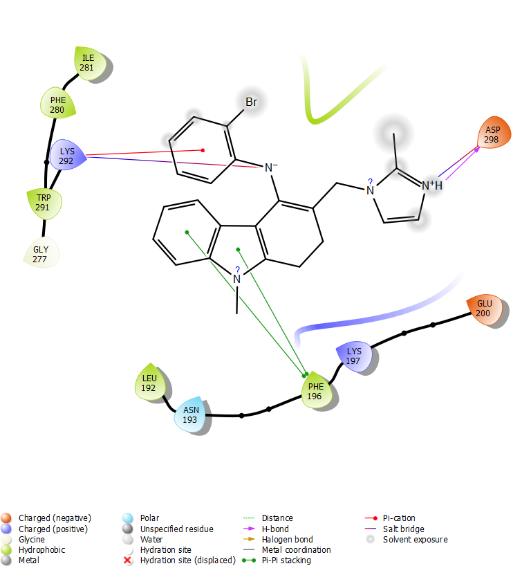 | 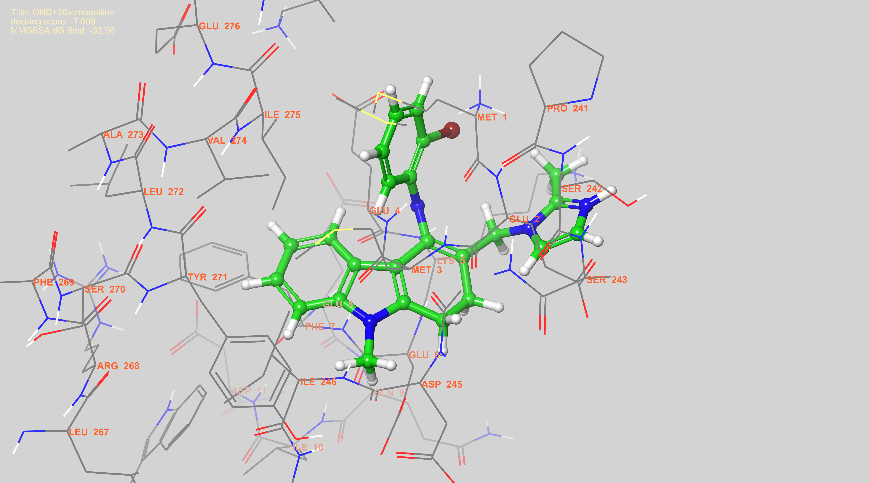 |
| 2D image & 3D images of FN-09 | |
| 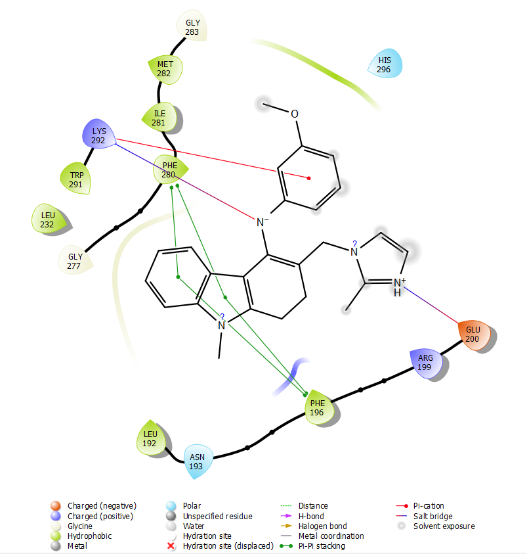 | 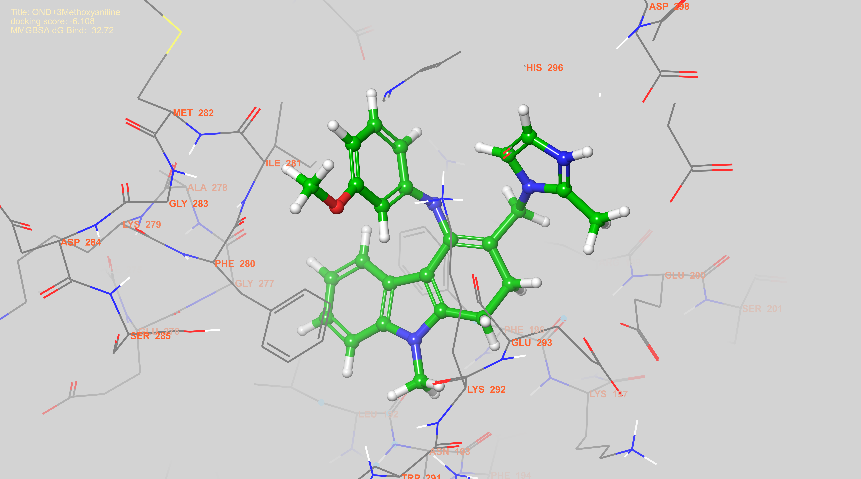 |
| 2D image & 3D images of FN-11 | |

- **Pharmacokinetic Profile of the selected compounds (ondansetron, FN-06 & FN-10)**

| 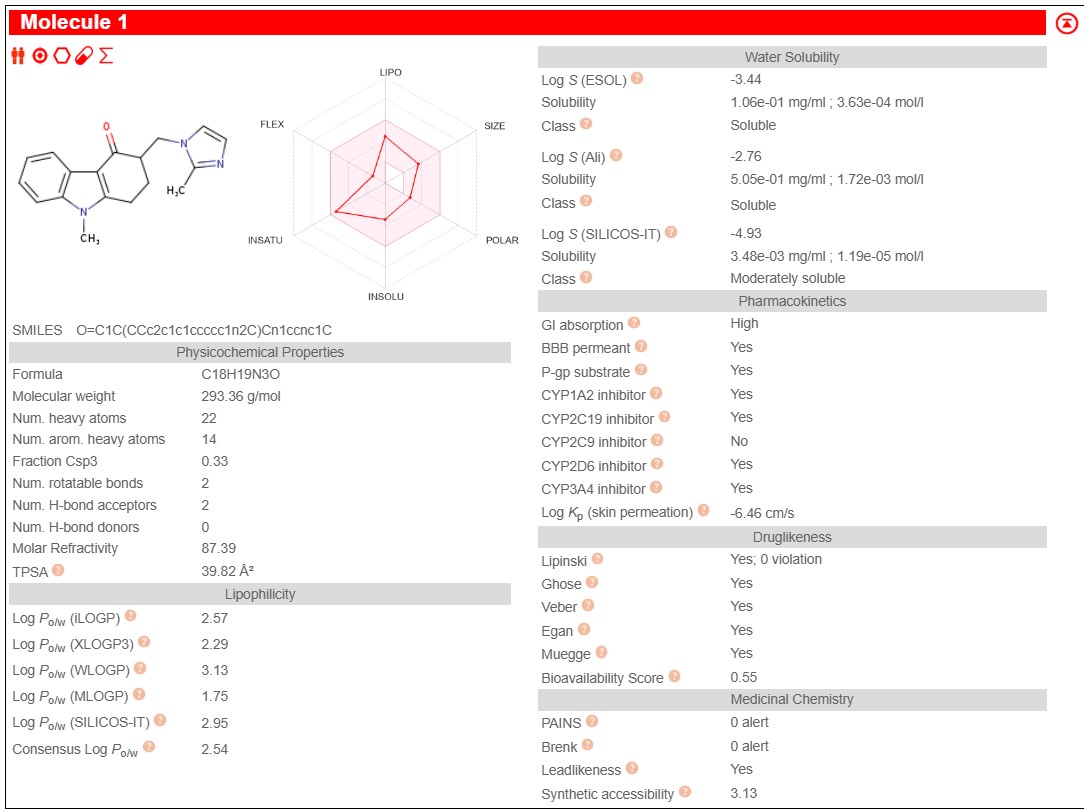 |
| --- |
| Profile of Ondansetron |
| 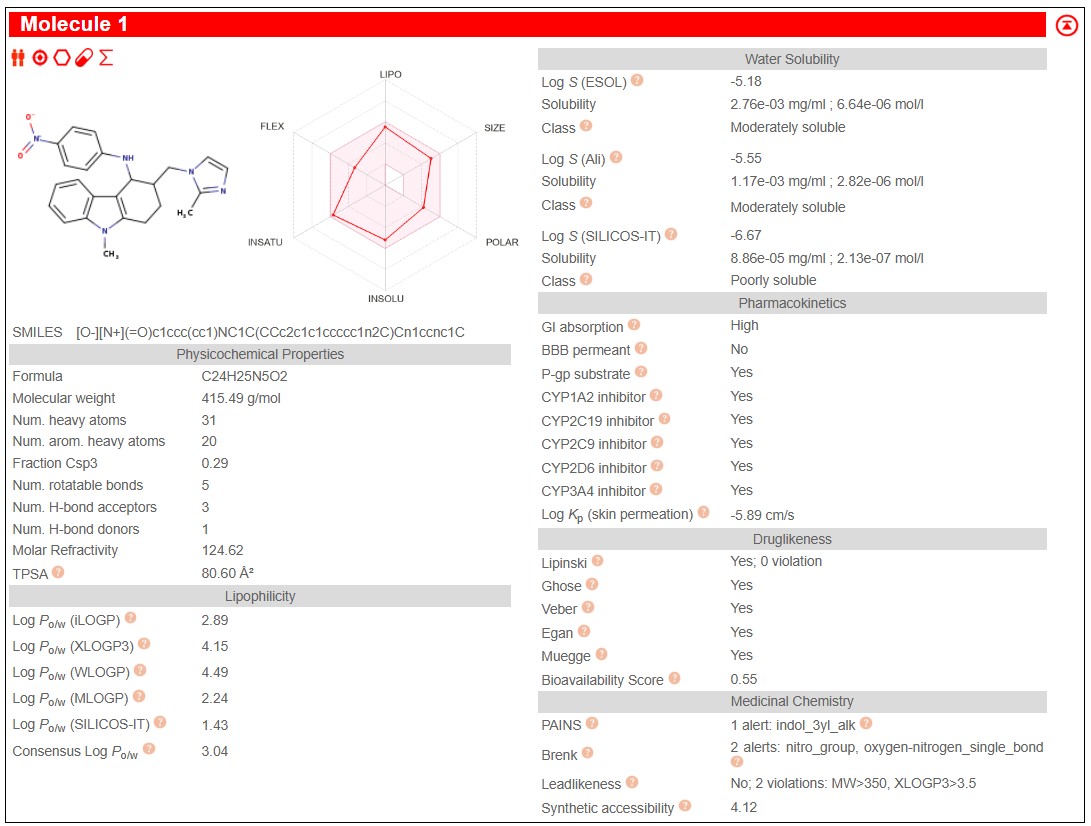 |
| Profile of FN-06 |
| 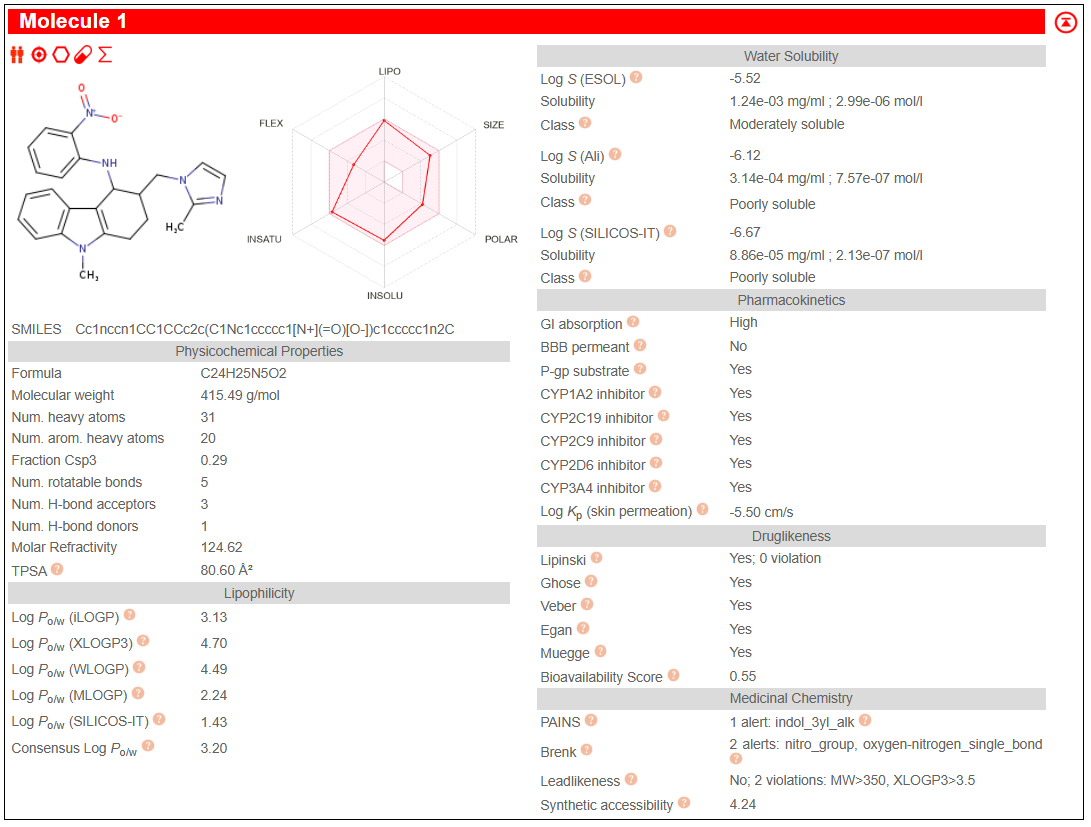 |
| Profile of FN-10 |

# Supplementary Data: Molecular Docking

Software: Maestro (Schrödinger Suite)
Force Field: OPLS4
Water Model: TIP3P

## 1**. Protein Preparation (Protein Preparation Wizard)**

| Parameter | Setting |
| --- | --- |
| Force field | OPLS4 |
| Add hydrogens | Yes |
| Missing loops/side chains | Filled using Prime |
| Water molecules | Removed beyond 5 Å from hetero groups |
| Protonation state | Epik at pH 7.0 ± 2.0 |
| H-bond optimization | Yes |
| Energy minimization | Restrained minimization (0.30 Å RMSD cutoff) |

## 2. Receptor Grid Generation

| Parameter | Setting |
| --- | --- |
| Grid center | Co-crystallized ligand centroid |
| Outer box size | 30 × 30 × 30 Å |
| Inner box size | 10 × 10 × 10 Å |
| Van der Waals scaling factor | 1.0 |
| Partial charge cutoff | 0.25 |
| Constraints | None (unless specified) |

## 3. Ligand Preparation (LigPrep)

| Parameter | Setting |
| --- | --- |
| Force field | OPLS4 |
| Ionization | Epik at pH 7.0 ± 2.0 |
| Tautomers | Generated |
| Stereoisomers | Up to 32 per ligand |
| Energy minimization | Yes |
| Desalting | Yes |

## 4. Docking Protocol (Glide)

| Parameter | Setting |
| --- | --- |
| Precision mode | SP or XP |
| Ligand sampling | Flexible |
| Nitrogen inversion | Allowed |
| Ring conformations | Sampled |
| Van der Waals scaling (ligand) | 0.80 |
| Partial charge cutoff (ligand) | 0.15 |
| Post-docking minimization | Yes |
| Number of poses retained | 10 per ligand |
| Pose ranking | GlideScore |

## 5. MM-GBSA Binding Energy Calculation (Prime)

| Parameter | Setting |
| --- | --- |
| Method | Prime MM-GBSA |
| Force field | OPLS4 |
| Solvent model | VSGB |
| Frames extracted | Every 10 ns |
| Entropy contribution | Not included (default) |

- **Data of Statistical Analysis (Invitro)**

| - **Table Analyzed First look grouped data** - **Two-way ANOVA Ordinary** - **Alpha 0.05** - **Source of Variation % of total variation P value P value summary Significant?** - **Interaction 1.977 <0.0001 **** Yes** - **Row Factor 97.48 <0.0001 **** Yes** - **Column Factor 0.4115 <0.0001 **** Yes** - **ANOVA table SS DF MS F (DFn, DFd) P value** - **Interaction 2919 18 162.2 F (18, 56) = 48.50 P<0.0001** - **Row Factor 143981 6 23997 F (6, 56) = 7175 P<0.0001** - **Column Factor 607.8 3 202.6 F (3, 56) = 60.58 P<0.0001** - **Residual 187.3 56 3.344** |
| --- |
| **Suramin Suramin Suramin Ondansetron(10mg/kg) Ondansetron(10mg/kg) Ondansetron(10mg/kg) FN-06(10mg/kg) FN-06(10mg/kg) FN-06(10mg/kg) FN-10(10mg/kg) FN-10(10mg/kg) FN-10(10mg/kg)**  **0 99.986 100.811 101.451 100.09 101.245 100.148 100.11 101.2 100.214 100.014 100.657 101.22**  **1 99.952 101.532 100.001 100.958 100.635 100.742 100.985 100.754 100.665 100.981 100.201 100.025**  **5 92.411 97.575 95.2 89.232 87.004 88.629 94.251 92.585 94.277 91.137 92.831 94.277**  **10 76.331 84.142 78.724 45.519 52.887 57.124 66.878 65.242 63.896 73.012 72.986 70.137**  **50 19.85 24.321 21.987 32.514 34.623 29.328 45.241 48.61 46.226 56.875 59.645 55.482**  **100 0 0 0 -2.33 0 0 0 -0.11 0 2.141 0 0**  **500 0 0 0 0 -2.01 -0.98 0 0 -0.01 0 -0.01 -0.01** |

- **Data of Statistical Analysis (Invivo)**
- **Data Blood glucose level**

**Saline(10mL/kg) Saline(10mL/kg) STZ(50mg/kg) STZ(50mg/kg) STZ(50mg/kg)+Ondansetron(10mg/kg) STZ(50mg/kg)+Ondansetron(10mg/kg) STZ(50mg/kg)+FN-10(10mg/kg) STZ(50mg/kg)+FN-10(10mg/kg) STZ(50mg/kg) +FN-06(10mg/kg) STZ(50mg/kg) +FN-06(10mg/kg) STZ(50mg/kg) + Metformin(500mg/kg) STZ(50mg/kg) + Metformin(500mg/kg)**

**1 105 107 117 130 201 210 184 189 196 187 139 123**

**6 95 99 200 210 192 196 134 139 168 161 105 110**

**12 70 72 269 277 168 172 110 115 156 151 99 97**

**18 100 101 299 310 155 158 92 96 100 96 89 85**

**24 97 96 366 371 101 105 69 72 88 81 70 80**

- **Test Blood glucose level**

**Within each row, compare columns (simple effects within rows)**

**Number of families 5**

**Number of comparisons per family 15**

**Alpha 0.05**

**Tukey's multiple comparisons test Mean Diff. 95.00% CI of diff. Significant? Summary Adjusted P Value**

**1**

**Saline(10mL/kg) vs. STZ(50mg/kg) -17.50 -32.00 to -2.998 Yes * 0.0110**

**Saline(10mL/kg) vs. Group C -99.50 -114.0 to -85.00 Yes **** <0.0001**

**Saline(10mL/kg) vs. STZ(50mg/kg)+FN-10(10mg/kg) -80.50 -95.00 to -66.00 Yes **** <0.0001**

**Saline(10mL/kg) vs. STZ(50mg/kg) +FN-06(10mg/kg) -85.50 -100.0 to -71.00 Yes **** <0.0001**

**Saline(10mL/kg) vs. Group F -25.00 -39.50 to -10.50 Yes *** 0.0002**

**STZ(50mg/kg) vs. Group C -82.00 -96.50 to -67.50 Yes **** <0.0001**

**STZ(50mg/kg) vs. STZ(50mg/kg)+FN-10(10mg/kg) -63.00 -77.50 to -48.50 Yes **** <0.0001**

**STZ(50mg/kg) vs. STZ(50mg/kg) +FN-06(10mg/kg) -68.00 -82.50 to -53.50 Yes **** <0.0001**

**STZ(50mg/kg) vs. Group F -7.500 -22.00 to 7.002 No ns 0.6217**

**Group C vs. STZ(50mg/kg)+FN-10(10mg/kg) 19.00 4.498 to 33.50 Yes ** 0.0049**

**Group C vs. STZ(50mg/kg) +FN-06(10mg/kg) 14.00 -0.5022 to 28.50 No ns 0.0633**

**Group C vs. Group F 74.50 60.00 to 89.00 Yes **** <0.0001**

**STZ(50mg/kg)+FN-10(10mg/kg) vs. STZ(50mg/kg) +FN-06(10mg/kg) -5.000 -19.50 to 9.502 No ns 0.8973**

**STZ(50mg/kg)+FN-10(10mg/kg) vs. Group F 55.50 41.00 to 70.00 Yes **** <0.0001**

**STZ(50mg/kg) +FN-06(10mg/kg) vs. Group F 60.50 46.00 to 75.00 Yes **** <0.0001**

**6**

**Saline(10mL/kg) vs. STZ(50mg/kg) -108.0 -122.5 to -93.50 Yes **** <0.0001**

**Saline(10mL/kg) vs. Group C -97.00 -111.5 to -82.50 Yes **** <0.0001**

**Saline(10mL/kg) vs. STZ(50mg/kg)+FN-10(10mg/kg) -39.50 -54.00 to -25.00 Yes **** <0.0001**

**Saline(10mL/kg) vs. STZ(50mg/kg) +FN-06(10mg/kg) -67.50 -82.00 to -53.00 Yes **** <0.0001**

**Saline(10mL/kg) vs. Group F -10.50 -25.00 to 4.002 No ns 0.2664**

**STZ(50mg/kg) vs. Group C 11.00 -3.502 to 25.50 No ns 0.2227**

**STZ(50mg/kg) vs. STZ(50mg/kg)+FN-10(10mg/kg) 68.50 54.00 to 83.00 Yes **** <0.0001**

**STZ(50mg/kg) vs. STZ(50mg/kg) +FN-06(10mg/kg) 40.50 26.00 to 55.00 Yes **** <0.0001**

**STZ(50mg/kg) vs. Group F 97.50 83.00 to 112.0 Yes **** <0.0001**

**Group C vs. STZ(50mg/kg)+FN-10(10mg/kg) 57.50 43.00 to 72.00 Yes **** <0.0001**

**Group C vs. STZ(50mg/kg) +FN-06(10mg/kg) 29.50 15.00 to 44.00 Yes **** <0.0001**

**Group C vs. Group F 86.50 72.00 to 101.0 Yes **** <0.0001**

**STZ(50mg/kg)+FN-10(10mg/kg) vs. STZ(50mg/kg) +FN-06(10mg/kg) -28.00 -42.50 to -13.50 Yes **** <0.0001**

**STZ(50mg/kg)+FN-10(10mg/kg) vs. Group F 29.00 14.50 to 43.50 Yes **** <0.0001**

**STZ(50mg/kg) +FN-06(10mg/kg) vs. Group F 57.00 42.50 to 71.50 Yes **** <0.0001**

**12**

**Saline(10mL/kg) vs. STZ(50mg/kg) -202.0 -216.5 to -187.5 Yes **** <0.0001**

**Saline(10mL/kg) vs. Group C -99.00 -113.5 to -84.50 Yes **** <0.0001**

**Saline(10mL/kg) vs. STZ(50mg/kg)+FN-10(10mg/kg) -41.50 -56.00 to -27.00 Yes **** <0.0001**

**Saline(10mL/kg) vs. STZ(50mg/kg) +FN-06(10mg/kg) -82.50 -97.00 to -68.00 Yes **** <0.0001**

**Saline(10mL/kg) vs. Group F -27.00 -41.50 to -12.50 Yes **** <0.0001**

**STZ(50mg/kg) vs. Group C 103.0 88.50 to 117.5 Yes **** <0.0001**

**STZ(50mg/kg) vs. STZ(50mg/kg)+FN-10(10mg/kg) 160.5 146.0 to 175.0 Yes **** <0.0001**

**STZ(50mg/kg) vs. STZ(50mg/kg) +FN-06(10mg/kg) 119.5 105.0 to 134.0 Yes **** <0.0001**

**STZ(50mg/kg) vs. Group F 175.0 160.5 to 189.5 Yes **** <0.0001**

**Group C vs. STZ(50mg/kg)+FN-10(10mg/kg) 57.50 43.00 to 72.00 Yes **** <0.0001**

**Group C vs. STZ(50mg/kg) +FN-06(10mg/kg) 16.50 1.998 to 31.00 Yes * 0.0185**

**Group C vs. Group F 72.00 57.50 to 86.50 Yes **** <0.0001**

**STZ(50mg/kg)+FN-10(10mg/kg) vs. STZ(50mg/kg) +FN-06(10mg/kg) -41.00 -55.50 to -26.50 Yes **** <0.0001**

**STZ(50mg/kg)+FN-10(10mg/kg) vs. Group F 14.50 -0.002165 to 29.00 No ns 0.0501**

**STZ(50mg/kg) +FN-06(10mg/kg) vs. Group F 55.50 41.00 to 70.00 Yes **** <0.0001**

**18**

**Saline(10mL/kg) vs. STZ(50mg/kg) -204.0 -218.5 to -189.5 Yes **** <0.0001**

**Saline(10mL/kg) vs. Group C -56.00 -70.50 to -41.50 Yes **** <0.0001**

**Saline(10mL/kg) vs. STZ(50mg/kg)+FN-10(10mg/kg) 6.500 -8.002 to 21.00 No ns 0.7478**

**Saline(10mL/kg) vs. STZ(50mg/kg) +FN-06(10mg/kg) 2.500 -12.00 to 17.00 No ns 0.9948**

**Saline(10mL/kg) vs. Group F 13.50 -1.002 to 28.00 No ns 0.0795**

**STZ(50mg/kg) vs. Group C 148.0 133.5 to 162.5 Yes **** <0.0001**

**STZ(50mg/kg) vs. STZ(50mg/kg)+FN-10(10mg/kg) 210.5 196.0 to 225.0 Yes **** <0.0001**

**STZ(50mg/kg) vs. STZ(50mg/kg) +FN-06(10mg/kg) 206.5 192.0 to 221.0 Yes **** <0.0001**

**STZ(50mg/kg) vs. Group F 217.5 203.0 to 232.0 Yes **** <0.0001**

**Group C vs. STZ(50mg/kg)+FN-10(10mg/kg) 62.50 48.00 to 77.00 Yes **** <0.0001**

**Group C vs. STZ(50mg/kg) +FN-06(10mg/kg) 58.50 44.00 to 73.00 Yes **** <0.0001**

**Group C vs. Group F 69.50 55.00 to 84.00 Yes **** <0.0001**

**STZ(50mg/kg)+FN-10(10mg/kg) vs. STZ(50mg/kg) +FN-06(10mg/kg) -4.000 -18.50 to 10.50 No ns 0.9576**

**STZ(50mg/kg)+FN-10(10mg/kg) vs. Group F 7.000 -7.502 to 21.50 No ns 0.6862**

**STZ(50mg/kg) +FN-06(10mg/kg) vs. Group F 11.00 -3.502 to 25.50 No ns 0.2227**

**24**

**Saline(10mL/kg) vs. STZ(50mg/kg) -272.0 -286.5 to -257.5 Yes **** <0.0001**

**Saline(10mL/kg) vs. Group C -6.500 -21.00 to 8.002 No ns 0.7478**

**Saline(10mL/kg) vs. STZ(50mg/kg)+FN-10(10mg/kg) 26.00 11.50 to 40.50 Yes **** <0.0001**

**Saline(10mL/kg) vs. STZ(50mg/kg) +FN-06(10mg/kg) 12.00 -2.502 to 26.50 No ns 0.1512**

**Saline(10mL/kg) vs. Group F 21.50 6.998 to 36.00 Yes ** 0.0012**

**STZ(50mg/kg) vs. Group C 265.5 251.0 to 280.0 Yes **** <0.0001**

**STZ(50mg/kg) vs. STZ(50mg/kg)+FN-10(10mg/kg) 298.0 283.5 to 312.5 Yes **** <0.0001**

**STZ(50mg/kg) vs. STZ(50mg/kg) +FN-06(10mg/kg) 284.0 269.5 to 298.5 Yes **** <0.0001**

**STZ(50mg/kg) vs. Group F 293.5 279.0 to 308.0 Yes **** <0.0001**

**Group C vs. STZ(50mg/kg)+FN-10(10mg/kg) 32.50 18.00 to 47.00 Yes **** <0.0001**

**Group C vs. STZ(50mg/kg) +FN-06(10mg/kg) 18.50 3.998 to 33.00 Yes ** 0.0064**

**Group C vs. Group F 28.00 13.50 to 42.50 Yes **** <0.0001**

**STZ(50mg/kg)+FN-10(10mg/kg) vs. STZ(50mg/kg) +FN-06(10mg/kg) -14.00 -28.50 to 0.5022 No ns 0.0633**

**STZ(50mg/kg)+FN-10(10mg/kg) vs. Group F -4.500 -19.00 to 10.00 No ns 0.9316**

**STZ(50mg/kg) +FN-06(10mg/kg) vs. Group F 9.500 -5.002 to 24.00 No ns 0.3700**

**Test details Mean 1 Mean 2 Mean Diff. SE of diff. N1 N2 q DF**

**1**

**Saline(10mL/kg) vs. STZ(50mg/kg) 106.0 123.5 -17.50 4.768 2 2 5.191 30.00**

**Saline(10mL/kg) vs. Group C 106.0 205.5 -99.50 4.768 2 2 29.51 30.00**

**Saline(10mL/kg) vs. STZ(50mg/kg)+FN-10(10mg/kg) 106.0 186.5 -80.50 4.768 2 2 23.88 30.00**

**Saline(10mL/kg) vs. STZ(50mg/kg) +FN-06(10mg/kg) 106.0 191.5 -85.50 4.768 2 2 25.36 30.00**

**Saline(10mL/kg) vs. Group F 106.0 131.0 -25.00 4.768 2 2 7.415 30.00**

**STZ(50mg/kg) vs. Group C 123.5 205.5 -82.00 4.768 2 2 24.32 30.00**

**STZ(50mg/kg) vs. STZ(50mg/kg)+FN-10(10mg/kg) 123.5 186.5 -63.00 4.768 2 2 18.69 30.00**

**STZ(50mg/kg) vs. STZ(50mg/kg) +FN-06(10mg/kg) 123.5 191.5 -68.00 4.768 2 2 20.17 30.00**

**STZ(50mg/kg) vs. Group F 123.5 131.0 -7.500 4.768 2 2 2.225 30.00**

**Group C vs. STZ(50mg/kg)+FN-10(10mg/kg) 205.5 186.5 19.00 4.768 2 2 5.636 30.00**

**Group C vs. STZ(50mg/kg) +FN-06(10mg/kg) 205.5 191.5 14.00 4.768 2 2 4.153 30.00**

**Group C vs. Group F 205.5 131.0 74.50 4.768 2 2 22.10 30.00**

**STZ(50mg/kg)+FN-10(10mg/kg) vs. STZ(50mg/kg) +FN-06(10mg/kg) 186.5 191.5 -5.000 4.768 2 2 1.483 30.00**

**STZ(50mg/kg)+FN-10(10mg/kg) vs. Group F 186.5 131.0 55.50 4.768 2 2 16.46 30.00**

**STZ(50mg/kg) +FN-06(10mg/kg) vs. Group F 191.5 131.0 60.50 4.768 2 2 17.94 30.00**

**6**

**Saline(10mL/kg) vs. STZ(50mg/kg) 97.00 205.0 -108.0 4.768 2 2 32.03 30.00**

**Saline(10mL/kg) vs. Group C 97.00 194.0 -97.00 4.768 2 2 28.77 30.00**

**Saline(10mL/kg) vs. STZ(50mg/kg)+FN-10(10mg/kg) 97.00 136.5 -39.50 4.768 2 2 11.72 30.00**

**Saline(10mL/kg) vs. STZ(50mg/kg) +FN-06(10mg/kg) 97.00 164.5 -67.50 4.768 2 2 20.02 30.00**

**Saline(10mL/kg) vs. Group F 97.00 107.5 -10.50 4.768 2 2 3.114 30.00**

**STZ(50mg/kg) vs. Group C 205.0 194.0 11.00 4.768 2 2 3.263 30.00**

**STZ(50mg/kg) vs. STZ(50mg/kg)+FN-10(10mg/kg) 205.0 136.5 68.50 4.768 2 2 20.32 30.00**

**STZ(50mg/kg) vs. STZ(50mg/kg) +FN-06(10mg/kg) 205.0 164.5 40.50 4.768 2 2 12.01 30.00**

**STZ(50mg/kg) vs. Group F 205.0 107.5 97.50 4.768 2 2 28.92 30.00**

**Group C vs. STZ(50mg/kg)+FN-10(10mg/kg) 194.0 136.5 57.50 4.768 2 2 17.05 30.00**

**Group C vs. STZ(50mg/kg) +FN-06(10mg/kg) 194.0 164.5 29.50 4.768 2 2 8.750 30.00**

**Group C vs. Group F 194.0 107.5 86.50 4.768 2 2 25.66 30.00**

**STZ(50mg/kg)+FN-10(10mg/kg) vs. STZ(50mg/kg) +FN-06(10mg/kg) 136.5 164.5 -28.00 4.768 2 2 8.305 30.00**

**STZ(50mg/kg)+FN-10(10mg/kg) vs. Group F 136.5 107.5 29.00 4.768 2 2 8.602 30.00**

**STZ(50mg/kg) +FN-06(10mg/kg) vs. Group F 164.5 107.5 57.00 4.768 2 2 16.91 30.00**

**12**

**Saline(10mL/kg) vs. STZ(50mg/kg) 71.00 273.0 -202.0 4.768 2 2 59.91 30.00**

**Saline(10mL/kg) vs. Group C 71.00 170.0 -99.00 4.768 2 2 29.36 30.00**

**Saline(10mL/kg) vs. STZ(50mg/kg)+FN-10(10mg/kg) 71.00 112.5 -41.50 4.768 2 2 12.31 30.00**

**Saline(10mL/kg) vs. STZ(50mg/kg) +FN-06(10mg/kg) 71.00 153.5 -82.50 4.768 2 2 24.47 30.00**

**Saline(10mL/kg) vs. Group F 71.00 98.00 -27.00 4.768 2 2 8.008 30.00**

**STZ(50mg/kg) vs. Group C 273.0 170.0 103.0 4.768 2 2 30.55 30.00**

**STZ(50mg/kg) vs. STZ(50mg/kg)+FN-10(10mg/kg) 273.0 112.5 160.5 4.768 2 2 47.61 30.00**

**STZ(50mg/kg) vs. STZ(50mg/kg) +FN-06(10mg/kg) 273.0 153.5 119.5 4.768 2 2 35.44 30.00**

**STZ(50mg/kg) vs. Group F 273.0 98.00 175.0 4.768 2 2 51.91 30.00**

**Group C vs. STZ(50mg/kg)+FN-10(10mg/kg) 170.0 112.5 57.50 4.768 2 2 17.05 30.00**

**Group C vs. STZ(50mg/kg) +FN-06(10mg/kg) 170.0 153.5 16.50 4.768 2 2 4.894 30.00**

**Group C vs. Group F 170.0 98.00 72.00 4.768 2 2 21.36 30.00**

**STZ(50mg/kg)+FN-10(10mg/kg) vs. STZ(50mg/kg) +FN-06(10mg/kg) 112.5 153.5 -41.00 4.768 2 2 12.16 30.00**

**STZ(50mg/kg)+FN-10(10mg/kg) vs. Group F 112.5 98.00 14.50 4.768 2 2 4.301 30.00**

**STZ(50mg/kg) +FN-06(10mg/kg) vs. Group F 153.5 98.00 55.50 4.768 2 2 16.46 30.00**

**18**

**Saline(10mL/kg) vs. STZ(50mg/kg) 100.5 304.5 -204.0 4.768 2 2 60.51 30.00**

**Saline(10mL/kg) vs. Group C 100.5 156.5 -56.00 4.768 2 2 16.61 30.00**

**Saline(10mL/kg) vs. STZ(50mg/kg)+FN-10(10mg/kg) 100.5 94.00 6.500 4.768 2 2 1.928 30.00**

**Saline(10mL/kg) vs. STZ(50mg/kg) +FN-06(10mg/kg) 100.5 98.00 2.500 4.768 2 2 0.7415 30.00**

**Saline(10mL/kg) vs. Group F 100.5 87.00 13.50 4.768 2 2 4.004 30.00**

**STZ(50mg/kg) vs. Group C 304.5 156.5 148.0 4.768 2 2 43.90 30.00**

**STZ(50mg/kg) vs. STZ(50mg/kg)+FN-10(10mg/kg) 304.5 94.00 210.5 4.768 2 2 62.44 30.00**

**STZ(50mg/kg) vs. STZ(50mg/kg) +FN-06(10mg/kg) 304.5 98.00 206.5 4.768 2 2 61.25 30.00**

**STZ(50mg/kg) vs. Group F 304.5 87.00 217.5 4.768 2 2 64.51 30.00**

**Group C vs. STZ(50mg/kg)+FN-10(10mg/kg) 156.5 94.00 62.50 4.768 2 2 18.54 30.00**

**Group C vs. STZ(50mg/kg) +FN-06(10mg/kg) 156.5 98.00 58.50 4.768 2 2 17.35 30.00**

**Group C vs. Group F 156.5 87.00 69.50 4.768 2 2 20.61 30.00**

**STZ(50mg/kg)+FN-10(10mg/kg) vs. STZ(50mg/kg) +FN-06(10mg/kg) 94.00 98.00 -4.000 4.768 2 2 1.186 30.00**

**STZ(50mg/kg)+FN-10(10mg/kg) vs. Group F 94.00 87.00 7.000 4.768 2 2 2.076 30.00**

**STZ(50mg/kg) +FN-06(10mg/kg) vs. Group F 98.00 87.00 11.00 4.768 2 2 3.263 30.00**

**24**

**Saline(10mL/kg) vs. STZ(50mg/kg) 96.50 368.5 -272.0 4.768 2 2 80.68 30.00**

**Saline(10mL/kg) vs. Group C 96.50 103.0 -6.500 4.768 2 2 1.928 30.00**

**Saline(10mL/kg) vs. STZ(50mg/kg)+FN-10(10mg/kg) 96.50 70.50 26.00 4.768 2 2 7.712 30.00**

**Saline(10mL/kg) vs. STZ(50mg/kg) +FN-06(10mg/kg) 96.50 84.50 12.00 4.768 2 2 3.559 30.00**

**Saline(10mL/kg) vs. Group F 96.50 75.00 21.50 4.768 2 2 6.377 30.00**

**STZ(50mg/kg) vs. Group C 368.5 103.0 265.5 4.768 2 2 78.75 30.00**

**STZ(50mg/kg) vs. STZ(50mg/kg)+FN-10(10mg/kg) 368.5 70.50 298.0 4.768 2 2 88.39 30.00**

**STZ(50mg/kg) vs. STZ(50mg/kg) +FN-06(10mg/kg) 368.5 84.50 284.0 4.768 2 2 84.24 30.00**

**STZ(50mg/kg) vs. Group F 368.5 75.00 293.5 4.768 2 2 87.05 30.00**

**Group C vs. STZ(50mg/kg)+FN-10(10mg/kg) 103.0 70.50 32.50 4.768 2 2 9.640 30.00**

**Group C vs. STZ(50mg/kg) +FN-06(10mg/kg) 103.0 84.50 18.50 4.768 2 2 5.487 30.00**

**Group C vs. Group F 103.0 75.00 28.00 4.768 2 2 8.305 30.00**

**STZ(50mg/kg)+FN-10(10mg/kg) vs. STZ(50mg/kg) +FN-06(10mg/kg) 70.50 84.50 -14.00 4.768 2 2 4.153 30.00**

**STZ(50mg/kg)+FN-10(10mg/kg) vs. Group F 70.50 75.00 -4.500 4.768 2 2 1.335 30.00**

**STZ(50mg/kg) +FN-06(10mg/kg) vs. Group F 84.50 75.00 9.500 4.768 2 2 2.818 30.00**
